# Global trends and disease burden of elderly male breast cancer, 1990-2021: a population-based study

**DOI:** 10.3389/fendo.2026.1674679

**Published:** 2026-02-06

**Authors:** Shaochun Liu, Jian Xu, Han Zhang, Yuhan Tang, Xiaoxi Han, Wenjing Xiong, Linlin Fan, Chang Su, Zhiqi Sui, Wenhui Zhao

**Affiliations:** 1 Department of Medical Oncology, Harbin Medical University Cancer Hospital, Harbin Medical University, Harbin, Heilongjiang, China; 2 Department of Oncology, the Second Affiliated Hospital of Anhui Medical University, Hefei, Anhui, China

**Keywords:** breast cancer, elderly male, global burden, incidence, mortality

## Abstract

**Objective:**

To assess global, regional, and national trends in the burden of elderly male breast cancer (EMBC) from 1990 to 2021 and to evaluate projected patterns to 2040.

**Methods:**

Estimates were obtained from the Global Burden of Disease 2021 study. Age-standardised incidence, mortality, and disability-adjusted life year rates (ASIR, ASMR, ASDR) were analysed across all countries and Sociodemographic Index (SDI) strata. Long-term changes were quantified using the average annual percent change derived from log-linear models. Joinpoint regression identified temporal inflection points. Age–period–cohort (APC) models characterise independent temporal effects. Mortality changes were decomposed into components attributable to population growth, ageing, and epidemiological change. Inequality was assessed using slope and concentration indexes. Attributable mortality and DALYs were evaluated for alcohol use, dietary risks, and tobacco. Future rates to 2040 were estimated using a Bayesian age–period–cohort (BAPC) model.

**Results:**

Globally, EMBC incidence, mortality, and DALYs increased from 1990 to 2021, with average annual percent changes(AAPC) of 1.8 (95% CI, confidence interval, 1.63 to 1.98), 0.58 (95% CI 0.38 to 0.77), and 0.68 (95% CI 0.43 to 0.92). East Asia recorded the steepest rise in incidence, increasing from 1.65 (95% UI, uncertainty interval, 1.16 to 2.42) to 6.65 per 100000 population (95% UI 2.77 to 9.78). The middle SDI quintile showed the largest increases in all three metrics, rising from 1.62 (95% UI 1.18 to 2.09) to 4.92 per 100000 population (95% UI 2.26 to 6.81). APC analysis indicated pronounced period and cohort effects in middle and low-middle SDI settings. Decomposition identified population growth as the dominant driver of rising burden. Alcohol use and dietary risks accounted for most increases in mortality and DALYs, while tobacco contributed minimally. Cross-country inequality was modest for incidence but more marked for mortality and DALYs. Projections suggest that age-standardised rates will decline gradually by 2040, although absolute case numbers may continue to rise in rapidly ageing regions.

**Conclusion:**

The global burden of EMBC continues to increase, with substantial regional and socioeconomic disparities. Although age-standardised rates are projected to decline, population ageing is expected to sustain or expand absolute numbers of cases and deaths.

## Introduction

Breast cancer arises from the malignant transformation of mammary epithelial cells and constitutes a leading contributor to global cancer morbidity and mortality. Classified primarily into invasive and non-invasive subtypes, it is closely linked to genetic susceptibility, hormonal exposure, and environmental determinants (1). As the most frequently diagnosed cancer worldwide, breast cancer also ranks as the leading cause of disability-adjusted life years (DALYs) across all malignancies (2).

Although male breast cancer (MBC) accounts for less than 1% of all newly diagnosed breast cancers (3), emerging evidence underscores its distinct biological behaviour and clinical manifestations compared with female breast cancer (FBC). Men typically present with larger tumour size, higher nodal involvement rates, and increased likelihood of distant metastasis at diagnosis (4), mainly attributable to delayed recognition of breast-related symptoms and the absence of gender-specific routine screening initiatives. Long-term Surveillance, Epidemiology, and End Results (SEER) data demonstrate that the age-adjusted incidence of MBC rose from 0.85 per 100,000 in 1975 to 1.43 in 2011, with a mean age at diagnosis of approximately 67 years (5). The global burden of MBC has steadily increased over recent decades (6).

This pattern reflects the rapid ageing of the global population. The risk of cancer increases markedly with advancing age (7). According to the WHO and the Lancet Commission on Ageing, individuals aged 60 years or older are internationally recognized as older adults (8). The World Health Organization projects that more than 20% of the global population will be aged 60 years or older by the mid-21st century, indicating that the cancer burden in older adults will continue to grow and contribute substantially to overall morbidity (9). The rising burden of cancer in this population poses a major public health challenge. It has direct implications for achieving Sustainable Development Goal 3.4, which targets reductions in premature mortality from non-communicable diseases through prevention and treatment (10).

In the present study, EMBC is explicitly defined as male breast cancer occurring in adults aged ≥60 years. Older men exhibit unique vulnerabilities, including a higher prevalence of BRCA2 mutations, greater multimorbidity burden, diminished physiological reserve, under-recognition of breast symptoms, and frequent misclassification as benign conditions such as gynecomastia (11, 12). Well-documented diagnostic delays in this population are associated with more advanced disease at presentation and poorer outcomes, highlighting the need for elderly-specific surveillance and intervention strategies beyond those developed for younger or all-age male populations.

Most epidemiological research on breast cancer has focused on women, and evidence specific to older men remains limited. Existing GBD-based assessments have described global male breast cancer patterns, yet they predominantly report all-age MBC and therefore obscure age-related risk structures, diagnostic pathways, and demographic pressures that shape the burden in older adults (13). The Global Burden of Disease (GBD) 2021 study offers a harmonised, methodologically consistent framework for evaluating these patterns across countries and over time (14). Within this context, a dedicated assessment of elderly male breast cancer (EMBC) is needed to determine whether temporal trajectories, regional disparities, and risk attributions diverge from those observed in younger men or in the male population as a whole, particularly as health systems confront the accelerating implications of population ageing.

To further elucidate the mechanisms underlying temporal change, the age-period-cohort (APC) model distinguishes biological ageing effects, period influences related to diagnostic or therapeutic evolution, and cohort-specific exposures shaped by generational context. This analytical structure provides a multidimensional interpretation of long-term incidence and mortality trends (15). Using GBD 2021 estimates, the present study applies APC modelling to characterise global, regional, and national trends in EMBC from 1990 to 2021, offering a comprehensive evaluation of its evolving burden in an ageing world.

## Methods

### Data source

Data for EMBC, defined as breast cancer occurring in men aged ≥60 years, were obtained from the GBD 2021 study. This study represents a secondary analysis of publicly available, de-identified GBD data. GBD 2021 provides harmonised estimates of incidence, mortality, and DALYs for 371 diseases and 88 risk factors across 204 countries and territories from 1990 to 2021. Estimates are generated using a hierarchical Bayesian meta-regression framework implemented in DisMod-MR 2.1, which incorporates epidemiological data, predictive covariates, and location-specific priors to produce internally consistent measures at global, regional, and national levels (16). The Sociodemographic Index (SDI), a composite indicator of income, education, and fertility, was used to stratify locations by development level (14). Detailed GBD query specifications, including cause codes, age groups, locations, extraction interface, and run date, are provided in the **Supplementary Methods**.

### Analysis of trends globally, five SDI quintiles, and 204 countries and territories

Age-standardized incidence (ASIR), mortality (ASMR), and disability-adjusted life year rates (ASDR) were evaluated from 1990 to 2021. Long-term temporal trends were quantified using the Average Annual Percent Change (AAPC), estimated through log-linear regression of annual standardized rates (17). Joinpoint regression was used to identify inflection points and to calculate annual percent change (APC) across detected segments (18). Model specifications, including the number of joinpoints tested, permutation procedures, and approaches to uncertainty estimation, are described in the **Supplementary Methods**.

### Age–period–cohort modelling

To assess the independent contributions of age, period, and cohort to observed temporal patterns, age–period–cohort models were fitted to GBD estimates. The selection of interval widths, reference categories, and the identifiability solution followed established methodological principles. The intrinsic estimator (IE) approach was applied to address the linear dependency among the three temporal dimensions (19). Details on model implementation, software versions, procedures for propagating GBD uncertainty intervals, and sensitivity analyses are provided in the **Supplementary Methods**.

### Decomposition analysis

Changes in EMBC mortality between 1990 and 2021 were decomposed into components attributable to population growth, population ageing, and changes in age-specific mortality rates. Interaction effects were also quantified to capture combined demographic and epidemiological influences (20). Full mathematical expressions and a numerical example illustrating component contributions are presented in the **Supplementary Methods**.

### Cross-country inequality analysis

Cross-national disparities in EMBC burden were evaluated using the slope index of inequality (SII) to quantify absolute inequality and the concentration index (CI) to quantify relative inequality. SII was estimated by regressing standardized outcomes on the SDI-based relative rank of each country, and CI was computed from the cumulative distribution of DALYs across ranked populations (21). Ranking procedures, weighting approaches, tie treatment, and statistical methods for uncertainty estimation are detailed in the **Supplementary Methods**.

### Risk factor analysis

Temporal changes in ASMR and ASDR attributable to dietary risks, alcohol use, and tobacco were quantified using the risk–outcome pairs defined in the GBD comparative risk assessment framework. This framework integrates exposure distributions, relative risks, and theoretical minimum risk exposure levels to estimate the burden attributable to modifiable risk factors across locations and years.

### Projection analysis

Projections of ASIR, ASMR, and ASDR for EMBC to 2040 were generated at the global level using a Bayesian age–period–cohort (BAPC) model (22). This approach applies Bayesian priors to stabilise age, period, and cohort effects and to propagate uncertainty, projecting future rates under the assumption that recently observed trends persist (23).

## Results

### Global trends

**Table 1**, **Supplementary Tables S1**–**S3** present the ASIR, ASMR, ASDR, and AAPC for EMBC across the global, five SDI quintiles, and 21 regions and 204 countries and territories in 1990 and 2021. The global trend analysis reveals that the ASIR, ASMR, and ASDR of EMBC all showed an increasing trend from 1990 to 2021, with AAPCs of 1.8 [95% CI, confidence interval, 1.63 to 1.98], 0.58 [95% CI 0.38 to 0.77], and 0.68 [95% CI 0.43 to 0.92], respectively. As shown in **Figure 1**, a global Joinpoint regression analysis identified a notable turning point in ASIR in 2013, with the APC dropping from 3.16 (2004–2013) to 0.24 (2013–2021). Similarly, both ASMR and ASDR experienced significant declines in 2013 and 2012, respectively, with APCs decreasing from 0.6 (2000–2013) to -0.4 (2013–2021) for ASMR and from 1.22 (2004–2012) to –0.29 (2012–2021) for ASDR.

**Table 1:** Incident cases and ASIR of EMBC in 1990 and 2021, and its temporal trends from 1990 to 2021.

Characteristics	1990	ASIR (95% UI)	2021	ASIR (95% UI)	1990-2021
	Incident cases (95% UI)		Incident cases (95% UI)		AAPC (95% CI)
Global	5798.25(5147.59-6686.47)	2.78(2.46-3.21)	23642.62(15832.48-29035.59)	4.72(3.2-5.77)	1.8 (1.63 to 1.98)
High SDI	2345.12(2177.31-2507.93)	3.96(3.66-4.24)	5619.03(5029.26-6217.35)	4.46(4-4.94)	0.35 (-0.35 to 1.05)
High-middle SDI	1341.54(1179.9-1540.82)	2.65(2.33-3.05)	6292.01(3949.46-8254.39)	5.46(3.49-7.12)	2.41 (2.16 to 2.67)
Low SDI	672.19(495.58-988.85)	5.52(4.06-8.21)	1549.35(1133.46-2445.57)	6.19(4.53-9.72)	0.38 (0.28 to 0.48)
Low-middle SDI	545.23(419.66-759.73)	1.69(1.29-2.38)	2416.1(1699.3-3103.72)	3.15(2.21-4.06)	2.06 (1.99 to 2.14)
Middle SDI	888.54(646.13-1154.37)	1.62(1.18-2.09)	7746.55(3486.78-10804.21)	4.92(2.26-6.81)	3.71 (3.36 to 4.07)
Region					
Andean Latin America	8.32(5.04-13.71)	0.74(0.45-1.22)	47.32(27.12-77.71)	1.4(0.8-2.3)	2.27 (1.7 to 2.85)
Australasia	58.06(40.86-81.08)	4.34(3.06-6.05)	155.54(103.15-221.18)	4.65(3.08-6.6)	0.65 (-1.09 to 2.42)
Caribbean	27.21(21.87-34.5)	1.82(1.46-2.31)	117.35(90.81-150.81)	3.8(2.94-4.88)	2.4 (0.84 to 3.99)
Central Asia	8.7(6.45-11.14)	0.44(0.32-0.57)	42.18(35.16-50.73)	1.13(0.95-1.35)	2.66 (0.67 to 4.7)
Central Europe	199.47(169.09-234.11)	2.6(2.21-3.06)	670.85(552.5-804.8)	5.36(4.42-6.43)	2.46 (1.94 to 2.98)
Central Latin America	30.1(27.35-32.99)	0.67(0.61-0.73)	194.92(164.18-231.38)	1.4(1.18-1.66)	2.51 (1.53 to 3.5)
Central Sub-Saharan Africa	43.94(24.32-77.77)	4(2.18-7.23)	107.32(61.15-196.09)	4.59(2.58-8.58)	0.44 (0.29 to 0.59)
East Asia	830.39(579.32-1199.78)	1.65(1.16-2.42)	9199.87(3738.58-13593.14)	6.65(2.77-9.78)	4.6 (4.24 to 4.96)
Eastern Europe	523.92(474.21-580.91)	4.58(4.14-5.08)	601.37(509.71-699.27)	3.42(2.9-3.98)	-0.85 (-1.92 to 0.24)
Eastern Sub-Saharan Africa	563.55(410.94-888.76)	14.49(10.51-23.11)	1307.51(840.86-2300.93)	16.84(10.89-29.46)	0.49 (0.45 to 0.52)
High-income Asia Pacific	110.11(82.68-147.55)	1.09(0.82-1.45)	388.55(278.15-520.32)	1.39(0.99-1.86)	0.63 (-0.32 to 1.58)
High-income North America	1439.24(1320.55-1556.34)	7.33(6.71-7.94)	2641.28(2405.82-2848.49)	6.53(5.94-7.05)	-0.24 (-1.04 to 0.56)
North Africa and Middle East	167.85(114.48-247.49)	1.84(1.25-2.7)	789.09(544.18-1130.99)	3.18(2.18-4.58)	1.82 (1.72 to 1.92)
Oceania	1.05(0.54-1.88)	0.66(0.35-1.18)	3.96(1.96-7.34)	0.97(0.49-1.8)	1.28 (0.92 to 1.63)
South Asia	474.74(351.18-661.42)	1.57(1.15-2.21)	2539.78(1497.1-3284.22)	3.19(1.89-4.12)	2.37 (1.98 to 2.77)
Southeast Asia	160.67(104.95-209.34)	1.31(0.85-1.71)	872.94(461.7-1168.36)	2.59(1.37-3.46)	2.24 (2.14 to 2.34)
Southern Latin America	80.05(58.26-110.09)	3.2(2.33-4.41)	201.95(147.7-274.33)	4.16(3.04-5.64)	0.98 (-1.34 to 3.36)
Southern Sub-Saharan Africa	31.84(23.19-46.01)	2.7(1.96-3.87)	115.74(79.21-149)	4.76(3.23-6.11)	1.83 (1.59 to 2.08)
Tropical Latin America	73.26(64.49-82.29)	1.49(1.3-1.67)	532.12(460.92-613.88)	3.69(3.19-4.26)	3.02 (1.87 to 4.19)
Western Europe	835.03(738.42-940.46)	2.75(2.43-3.1)	2851.15(2347.16-3417.45)	5.14(4.24-6.16)	2.13 (1.45 to 2.82)
Western Sub-Saharan Africa	130.75(75.43-201.01)	2.83(1.61-4.3)	261.82(166.09-462.51)	2.88(1.78-4.96)	0.05 (-0.02 to 0.12)

ASIR, age-standardized incidence rate; EMBC, Elderly male breast cancer; AAPC, average annual percentage change; UI, uncertainty interval; CI, confidence interval.

**Figure 1:**
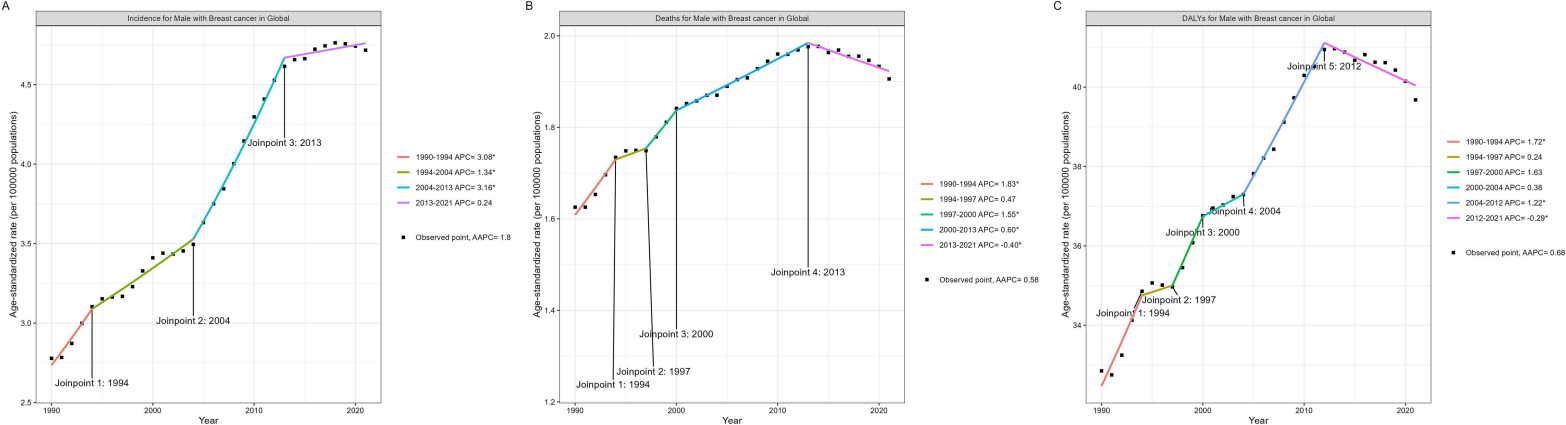
Joinpoint regression analysis of EMBC for ASIR **(A)**, ASMR **(B)**, and ASDR **(C)**, 1990–2021. Age definition: EMBC refers to men aged ≥60 years. Age standardization: Standardized to the GBD 2017 world standard population. Unit: per 100,000 population. EMBC, elderly male breast cancer; ASIR, age-standardized incidence rate; ASMR, age-standardized mortality rate; ASDR, age-standardized disability-adjusted life years; APC, annual percent change; AAPC, average annual percent change.

### Trends across the five SDI quintiles

ASIR increased across all SDI quintiles. The most notable increase occurred in the middle SDI quintile, where it rose from 1.62 per 100,000 population (95% UI, uncertainty interval, 1.18 to 2.09) in 1990 to 4.92 per 100,000 population (95% UI 2.26–6.81) in 2021, with an AAPC of 3.71 (95% CI 3.36 to 4.07). Similarly, the most significant increases in ASMR and ASDR were observed in the middle SDI quintile, with AAPCs of 1.56 (95% CI 1.35 to 1.78) and 1.75 (95% CI 1.56 to 1.93), respectively. In contrast, the smallest increase in ASIR was seen in the high SDI quintile (AAPC 0.35 [95% CI –0.35 to 1.05]), and the high SDI quintile showed the most pronounced declines in ASDR and ASMR among the five SDI quintiles, with AAPCs of –0.47 (95% CI –0.99 to 0.06) and –0.37 (95% CI –0.93 to 0.2).

### Trends across 21 regions and 204 countries and territories

By region, East Asia recorded the highest ASIR in 2021, whereas Eastern Sub-Saharan Africa had the highest ASMR and ASDR. Between 1990 and 2021, East Asia also experienced the most pronounced increase in ASIR, rising from 1.65 per 100,000 population (95% UI 1.16 to 2.42) in 1990 to 6.65 per 100,000 population (95% UI 2.77 to 9.78) in 2021, with an AAPC of 4.6 (95% CI 4.24 to 4.96). The ASMR and ASDR in Eastern Sub-Saharan Africa reached 14.41 per 100,000 population (95% UI 9.41 to 25.39) and 269.29 per 100,000 population (95% UI 174.93 to 476.59), respectively. In contrast, the ASIR in Western Sub-Saharan Africa showed no significant temporal change, with an AAPC of 0.05 (95% CI to0.02 to 0.12).

At the country level, Uganda had the highest ASIR and ASMR for elderly male breast cancer in both 1990 and 2021. The ASIR was 17.85 per 100,000 population in 1990 (95% UI 9.43 to 30.75) and increased to 24.98 per 100,000 population in 2021 (95% UI 12.48 to 45.27). The ASMR was 16.87 per 100,000 population in 1990 (95% UI 8.83 to 29.1) and reached 21.17 per 100,000 population in 2021 (95% UI 10.65 to 37.55). The country with the highest ASDR in 2021 was Malawi, with a rate of 308.26 per 100,000 population (95% UI 130.87 to 603.06).

**Figure 2:**
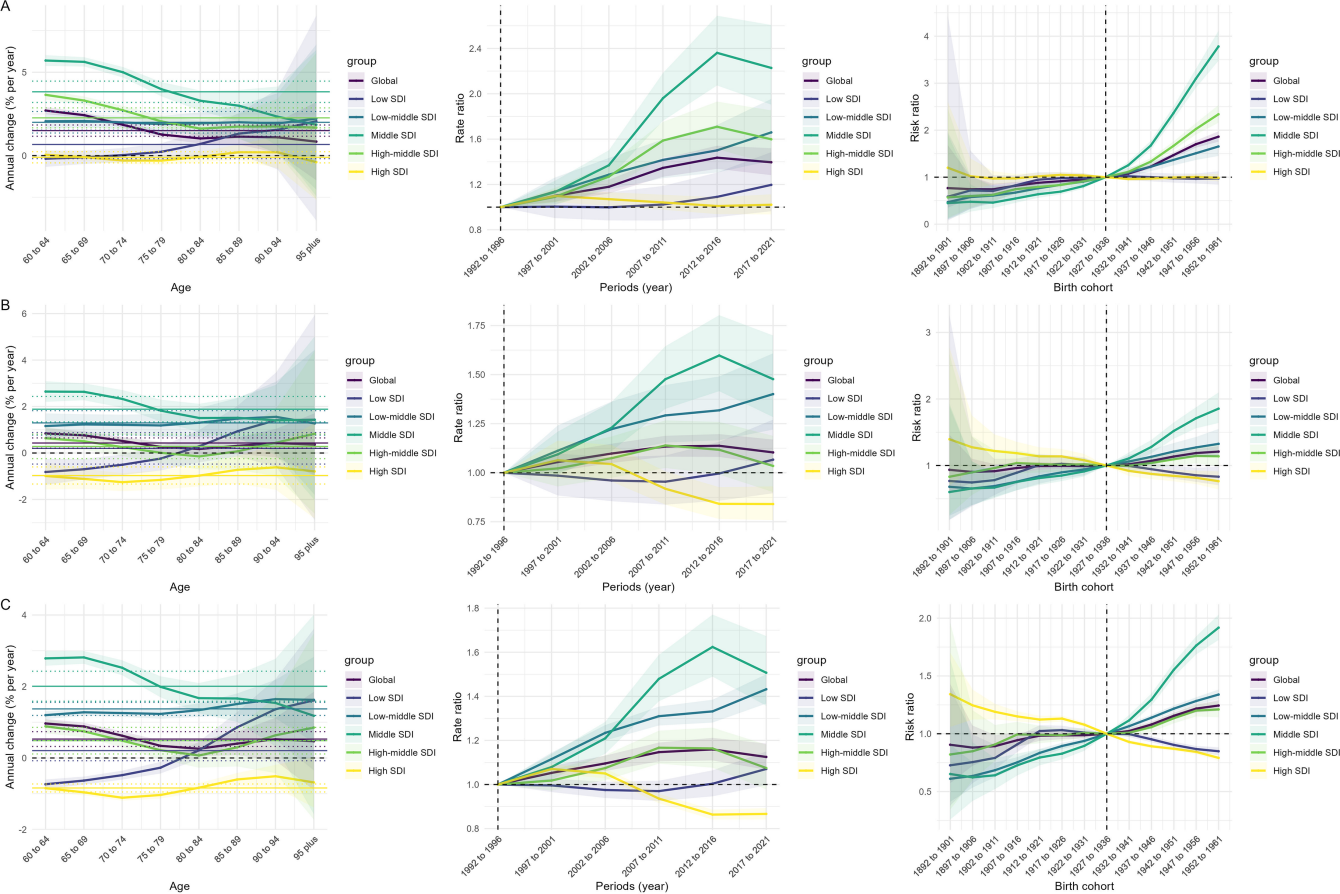
Global map of ASIR of EMBC in 1990 **(A)** and 2021 **(B)**, and corresponding AAPC from 1990 to 2021 **(C)**. Age definition: EMBC refers to men aged ≥60 years. Age standardization: Age-standardized to the GBD 2017 world standard population. Unit: per 100,000 population. EMBC, elderly male breast cancer; ASIR, age-standardized incidence rate; AAPC, average annual percentage change.

**Figure 2** presents the differences in ASIR and the corresponding AAPCs across countries and regions in 1990 and 2021.

### Age-period-cohort analysis on ASIR, ASMR, and ASDR

**Figure 3:**
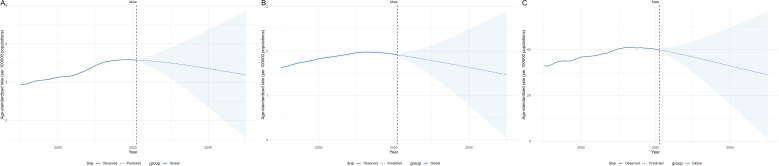
Age–period–cohort effects on incidence **(A)**, mortality **(B)**, and DALYs **(C)** of EMBC globally and by SDI level. Age definition: EMBC refers to men aged ≥60 years. Unit: per 100,000 population. EMBC, elderly male breast cancer; SDI, Sociodemographic Index; DALYs, disability-adjusted life years.

**Figure 3** summarises the age, period and cohort patterns of incidence, mortality and DALYs for EMBC at the global level and across SDI categories. The incidence results show small positive local drifts in the younger elderly groups and values close to zero or slightly negative in advanced ages. The period effects show consistent increases, and the cohort effects rise sharply in later birth cohorts, particularly in middle- and low-middle-SDI regions. In contrast, the mortality and DALYs results show uniformly negative local drifts across ages, modest period increases in most low-SDI settings and cohort effects that decline towards the mid-century birth cohorts before levelling off or showing only mild recovery in later cohorts. High-SDI regions display the most stable incidence drifts, the smallest period increases and cohort patterns that remain close to unity for incidence and below unity for both mortality and DALYs.

### Decomposition analysis

**Figure 4:**
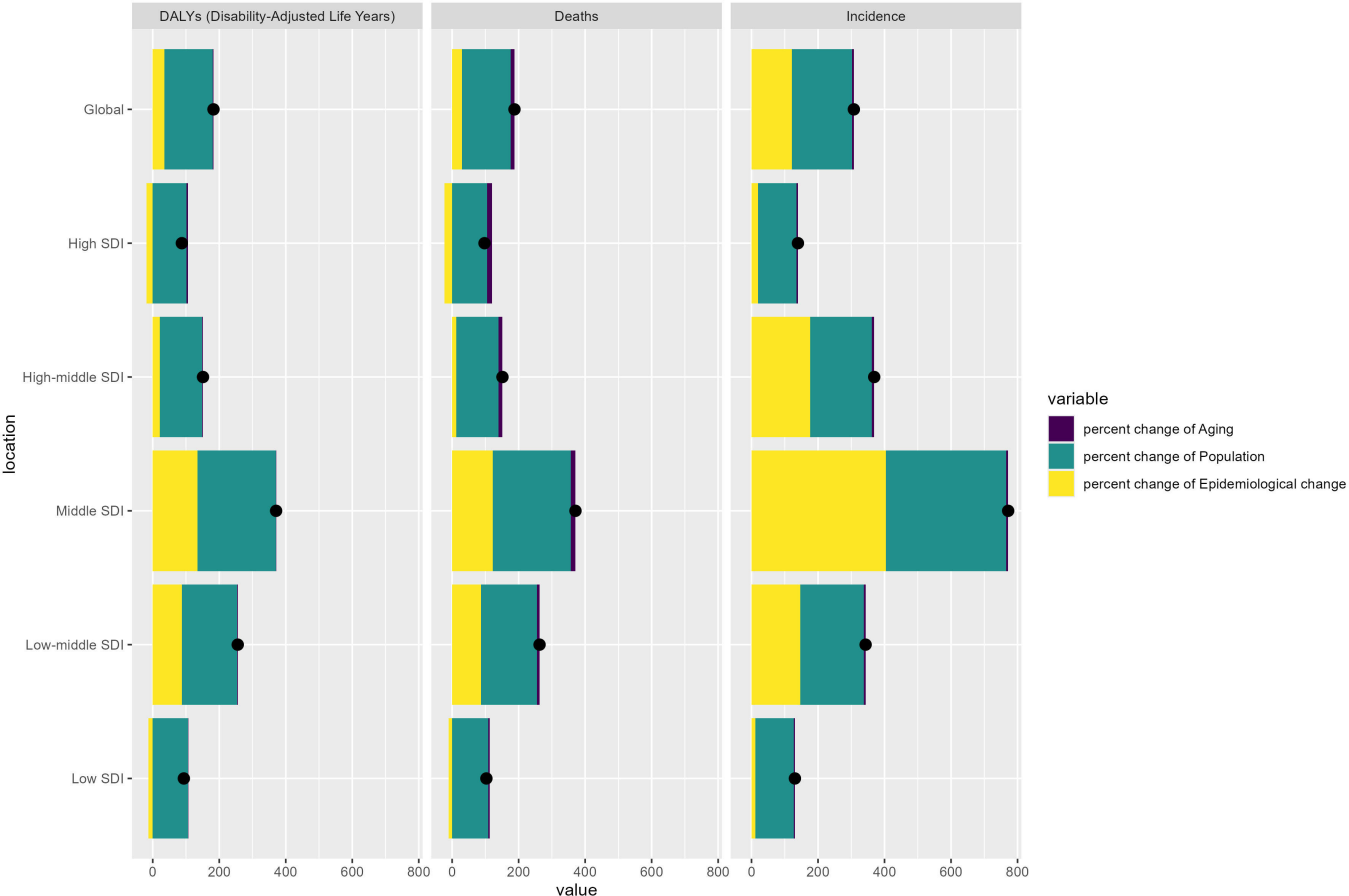
Decomposition of changes in incidence, mortality, and DALYs of EMBC from 1990 to 2021 by SDI quintile. DALYs, disability-adjusted life years; SDI, Sociodemographic Index.

**Figure 4** presents the decomposition analysis of ASIR, ASMR, and ASDR for breast cancer in older males across different SDI quintiles, highlighting the roles of aging, population growth, and epidemiological changes. Regarding ASIR, epidemiological changes exerted a greater influence than ASMR and ASDR, particularly in the middle and low-middle SDI quintiles. Population growth remained the predominant driver of increases in these regions, while the effect of aging remained relatively limited across all SDI levels. For ASMR, population growth emerged as the primary contributor, with the most pronounced changes observed in the middle and low SDI quintiles. The influence of aging was most significant in the high SDI quintile, though its effect was less notable elsewhere. The impact of epidemiological changes varied, with marked contributions seen in the middle SDI quintile.

Regarding ASDR, population growth accounted for the largest share of the increase across all SDI quintiles, particularly in middle- and low-SDI areas. The influence of aging was more prominent in the high and high-middle SDI quintiles. At the same time, the effects of epidemiological changes were generally modest, with some increase seen in the middle and low SDI quintile. In summary, the decomposition analysis underscores that population growth is the leading factor behind the rising breast cancer burden in older males, with aging playing a more prominent role in higher SDI quintiles and epidemiological changes contributing more variably, especially in middle and low SDI quintiles. **Supplementary Tables S4**–**S6** detail the changes in incidence, deaths, and DALYs attributed to population-level determinants and causes from 1990 to 2021.

### Cross-country inequality analysis

**Figure 5:**
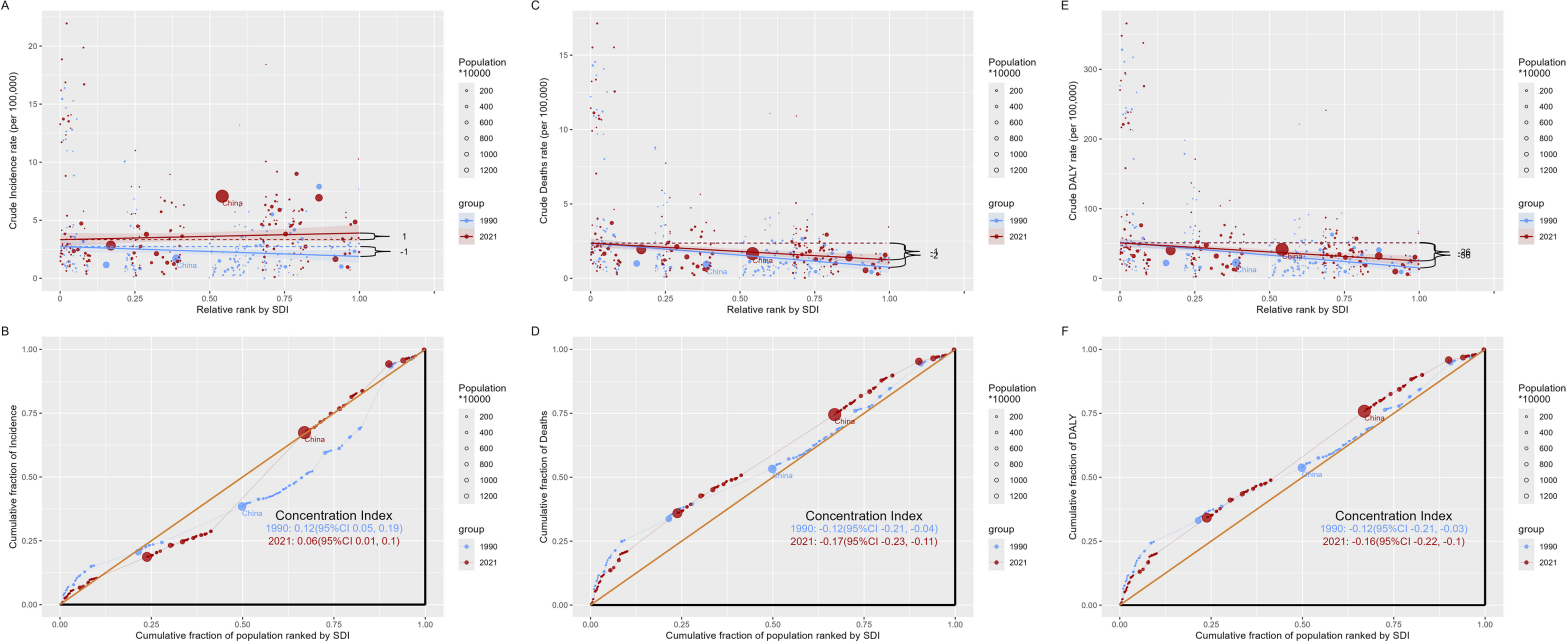
SDI-related inequality in ASIR, ASMR, and ASDR of EMBC in 1990 and 2021: regression-based slope index of inequality **(A, C, E)** and concentration curves **(B, D, F)**. Age definition: EMBC refers to men aged ≥60 years. Age standardization: Age-standardized to the GBD 2017 world standard population. Unit: per 100,000 population. EMBC, elderly male breast cancer; ASIR, age-standardized incidence rate; ASMR, age-standardized mortality rate; ASDR, age-standardized disability-adjusted life years; SDI, Sociodemographic Index.

There is a degree of absolute and relative inequality in SDI observed in breast cancer burden, although it is not particularly significant (**Figure 5**). The differences in the concentration index, which measures relative gradient inequality, between 1990 and 2021 show that while the concentration index for ASIR was 0.12 (95% CI: 0.05–0.19) in 1990 and decreased to 0.06 (95% CI: 0.01–0.10) in 2021, indicating a narrowing imbalance in the distribution of incidence rates across countries with different SDI, the inequality remains. In contrast, the concentration index for ASMR was -0.12 (95% CI: -0.21 to -0.04) in 1990 and -0.17 (95% CI: -0.23 to -0.11) in 2021, while the concentration index for ASDR was -0.12 (95% CI: -0.21 to -0.03) in 1990 and -0.16 (95% CI: -0.22 to -0.10) in 2021. These findings suggest that the distribution of mortality and DALYs burden across countries with different SDIs shows an increasing imbalance.

### Risk factor analysis

**Figure 6:**
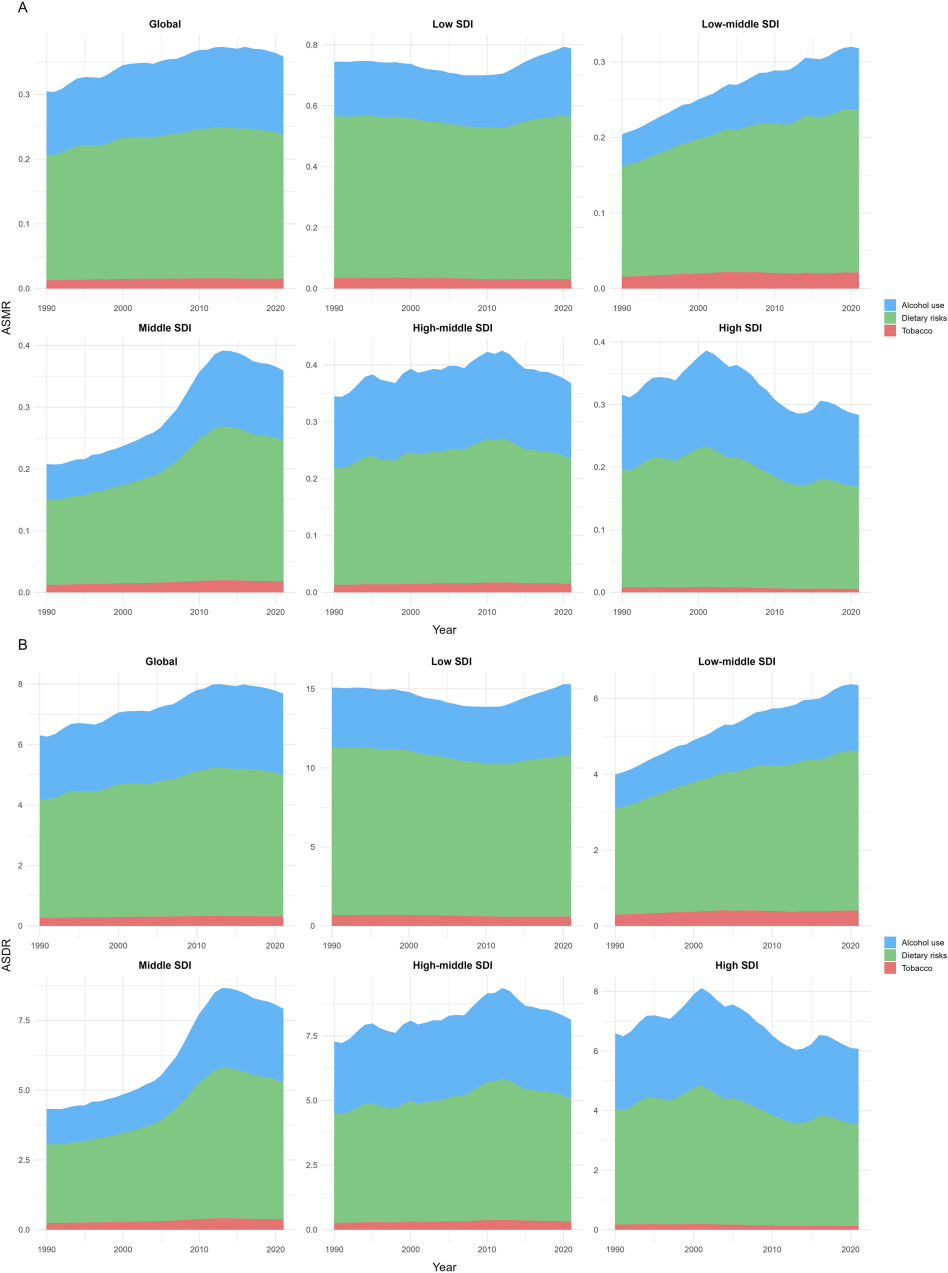
Temporal trends in ASMR **(A)** and ASDR **(B)** of EMBC attributable to alcohol use, dietary risks, and tobacco across SDI quintiles and globally, 1990–2021. Age definition: EMBC refers to men aged ≥60 years. Age standardization: Age-standardized to the GBD 2017 world standard population. Unit: per 100,000 population. EMBC, elderly male breast cancer; ASMR, age-standardized mortality rate; ASDR, age-standardized disability-adjusted life years; SDI, Sociodemographic Index.

**Figure 6** depicts the temporal trends in ASMR and ASDR for EMBC from 1990 to 2021, attributable to three principal risk factors at both global and regional levels. Globally, alcohol consumption emerged as the foremost contributor to increased ASMR and ASDR, with dietary risks following as the second most significant factor. From 1990 to 2015, the burden linked to alcohol and dietary risks steadily escalated before declining between 2015 and 2021.

At the SDI quintile level, the low SDI quintile initially experienced a gradual decline in the impact of alcohol and dietary factors on ASMR and ASDR from 1990, only to see a modest resurgence around 2010. In contrast, the low‐middle quintile exhibited a continuous rise in alcohol‐driven ASMR and ASDR throughout the period, while the middle quintile showed an upward trend from 1990 that reversed in the early 2010s. The high‐middle quintile maintained relative stability from 1990 to 2021, and in the high quintile, ASMR and ASDR increased from 1990 before gradually declining in the 21st century. Notably, tobacco use had the least impact across all quintiles, although a slight upward trend was observed in the low‐middle and middle SDI groups.

### BAPC prediction

**Figure 7:**
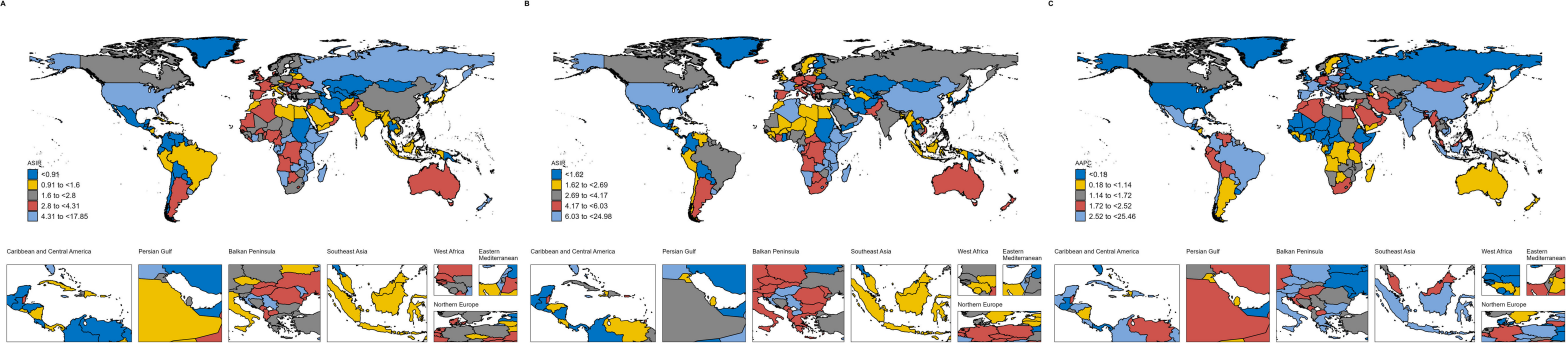
Observed and projected global ASIR **(A)**, ASMR **(B)**, and ASDR **(C)** of EMBC, 1990–2040. Age definition: EMBC refers to men aged ≥60 years. Age standardization: Age-standardized to the GBD 2017 world standard population. Unit: per 100,000 population. EMBC, elderly male breast cancer; ASIR, age-standardized incidence rate; ASMR, age-standardized mortality rate; ASDR, age-standardized disability-adjusted life years.

**Figure 7** shows that the projected global burden of EMBC declines steadily after 2021. By 2040, the predicted ASIR, ASMR, and ASDR for EMBC are 4.06 (95% UI 1.45–7.08), 1.63 (95% UI 0.77–2.49), and 32.85 (95% UI 16.15–49.55), respectively.

## Discussion

This study provides the first comprehensive global assessment of EMBC from 1990 to 2021. The results show that EMBC follows a temporal trajectory that is distinct from both all-age MBC and female breast cancer. Incidence, mortality and DALYs rose steadily for over two decades and then slowed after 2012–2013, consistent with the turning points detected in Joinpoint analyses. This synchronous rise contrasts with the global pattern in female breast cancer, where incidence has continued to rise while mortality has declined steadily over the same period (24–26). They also differ from all-age MBC, which generally shows an earlier separation between rising incidence and declining mortality (13). This distinction confirms the added value of restricting analysis to older men and demonstrates that EMBC cannot be inferred from existing all-age estimates.

The unique epidemiology of EMBC likely reflects the intersection of ageing biology, male-specific risk profiles and health system limitations. Older men have a higher prevalence of BRCA2 mutations, multimorbidity and diminished physiologic reserve, and they commonly experience diagnostic delays because breast symptoms are often misattributed to benign causes such as gynecomastia (11, 12, 27). These factors contribute to more advanced stage at diagnosis and may explain why mortality trends in EMBC mirror incidence more closely than in female breast cancer, where long-standing investment in screening and treatment has produced substantial survival gains (25, 26).

Marked geographic heterogeneity was observed. East Asia shows the highest incidence in 2021 and the steepest long-term increase, which corresponds with rapid population ageing, lifestyle transitions, and improved registry completeness in the region (28, 29). At the same time, several African countries exhibit the highest mortality and DALYs, which likely reflects persistent barriers to diagnosis, limited pathology infrastructure, delayed presentation and constrained access to therapy. These findings align with broader evidence that many cancers in Africa continue to show limited improvements in survival despite global progress (30).

High SDI regions demonstrated earlier declines in incidence, mortality, and DALYs than other SDI quintiles. This may reflect stabilised exposure to major risk factors, broader access to diagnostic imaging and therapeutic advances. However, older men with breast cancer benefit less from endocrine therapy and targeted agents such as CDK4/6 inhibitors compared with younger patients because multimorbidity, frailty and drug tolerance frequently limit treatment intensification (6, 12). Competing risks from cardiovascular disease, diabetes, and chronic pulmonary conditions also attenuate the mortality reductions that might otherwise accompany therapeutic advances (31).

Age–period–cohort analysis provides additional insight into these patterns. Incidence increases most consistently across younger elderly groups and in middle and low-middle SDI settings, suggesting that later birth cohorts may have accumulated higher lifetime risk. The rise in cohort risk after 1962 mirrors patterns observed for hormone receptor positive female breast cancer in Scotland and elsewhere (32). Period effects demonstrate broad global increases, which likely reflect enhanced diagnostic access and improved cancer registration, particularly in evolving health systems across Asia and Latin America (29). Mortality and DALYs show negative local drifts across many age groups, although gains are not as marked as those observed for women.

The decomposition analysis highlights population growth as the dominant driver of the global rise in EMBC, particularly in middle- and low-SDI regions. Ageing contributes more substantially in high SDI regions. Epidemiological change plays a varying role but is most pronounced in settings undergoing rapid urbanisation and metabolic transition, which mirrors patterns observed in colorectal cancer and pancreatic cancer among older adults (31, 33).

Although cross-country inequality remained modest in absolute terms, its direction differed across indicators. Consistent with our inequality analysis, incidence inequality narrowed slightly from 1990 to 2021. In contrast, mortality and DALYs became increasingly concentrated in lower SDI countries, reflecting a widening fatal burden in settings with limited diagnostic and treatment capacity. These patterns align with evidence that cancer survival improvements have disproportionately benefited higher-SDI regions, while lower-resource countries continue to experience persistently higher mortality despite similar incidence levels (30, 34). Without targeted strategies, these disparities are likely to grow as populations age.

The interpretation of risk factors requires caution because most etiologic evidence for breast cancer originates from studies in women, whereas research in older men remains scarce. In our analysis, alcohol and dietary risks were the predominant contributors to EMBC mortality and DALYs, increasing steadily from 1990 to 2015 before declining modestly thereafter. In contrast, tobacco consistently accounted for the smallest proportion and showed only slight increases in low-middle and middle SDI regions (13). These temporal patterns align with findings from multinational pooling studies of male breast cancer, which report modest but significant associations between alcohol intake and cancer risk, with odds ratios typically ranging from 1.2 to 1.8 for moderate-to-high consumption (35, 36). In contrast, female breast cancer meta-analyses demonstrate a more gradual dose–response relationship, with risk increasing by approximately 7 to 10 percent per 10 g/day of alcohol and reaching relative risks of 1.3 to 1.5 only at higher intake levels (37). Beyond differences in effect magnitude, underlying mechanisms may diverge between sexes: in men, alcohol-related carcinogenesis appears more strongly linked to hepatic dysfunction and impaired oestrogen clearance, whereas studies in women emphasise direct hormonal pathways (35). The regional heterogeneity in our results, including sharper alcohol- and diet-related burdens in middle and low-middle SDI settings, likely reflects variation in exposure patterns, metabolic profiles and health system resources. These discrepancies underscore the uncertainty in extrapolating evidence from female-dominated studies to EMBC and highlight the need for male-specific epidemiologic research. The prominent role of alcohol and metabolic risks observed in our findings supports integrated non-communicable disease prevention strategies tailored to ageing male populations.

Based on projections from the BAPC model, the global burden of EMBC is expected to decline, a pattern that mirrors forecasts for all-age male breast cancer. In clear contrast, the projected incidence and mortality of female breast cancer continue to rise through 2050 (38). This divergence likely reflects the influence of multiple risk factors that drive female breast cancer globally, with shifting exposure patterns compounded by demographic transitions that enlarge the pool of individuals at risk (39). Concurrently, delayed childbearing, rising obesity and metabolic risk, increasing breast density, and accumulated hormone-related exposures in several countries further reinforce the sustained upward trajectory of female breast cancer (24). It is important to note that although age-standardised rates of EMBC are declining, the absolute number of cases and deaths may continue to increase in regions experiencing rapid population ageing, particularly in East Asia, Eastern Europe, and several middle-to-high SDI settings where the population of older men is expanding substantially (40).

Diagnostic improvements may also explain portions of the observed temporal changes. Increased utilisation of breast imaging modalities among high-risk men, including mammography, ultrasound and MRI, has enhanced case detection and contributed to greater awareness of male breast disease in clinical practice (41). Therapeutic advances in endocrine therapy, chemotherapy, and targeted agents have improved outcomes for some older men, although treatment limitations related to frailty and polypharmacy persist (5). Older patients face substantial competing mortality from cardiovascular disease, diabetes, chronic respiratory diseases, and frailty-related conditions that may attenuate survival gains even when cancer control improves (42).

This study has several limitations. First, GBD estimates depend on statistical modelling in data-sparse settings where registration systems are incomplete, which may influence regional comparisons (39). Second, the ecological association between SDI and EMBC outcomes carries inherent limitations and may not capture individual-level risk. Third, GBD does not include cancer stage, molecular subtype or treatment information, which limits interpretation of age-specific pathways. Fourth, independent validation using data sources such as SEER is limited for elderly male populations. Fifth, the large number of comparisons across SDI levels, regions and countries increases the likelihood of false-positive findings. Although consistent direction of patterns and sensitivity analyses mitigate this concern, caution is warranted when interpreting marginal differences. Finally, uncertainty intervals around incidence, mortality and DALYs should be considered when comparing country ranks and regional contrasts.

## Conclusion

In conclusion, EMBC displays a distinct epidemiologic trajectory, marked by long-term increases in ASIR, ASMR, and ASDR and emerging evidence of recent stabilisation in selected high-SDI settings. BAPC projections indicate a gradual decline in global ASIR, ASMR, and ASDR through 2040. Persistent excess mortality and DALYs in low-SDI countries reflect constrained diagnostic capacity, delayed presentation, and limited access to systemic therapy. Alcohol and dietary risks remain the dominant contributors to the attributable burden, while cohort patterns point to heightened susceptibility among generations born after the early 1960s. These findings underscore an urgent need for region-tailored strategies that include awareness initiatives for older men, integration of context-appropriate early detection approaches, strengthened pathology infrastructure, and equitable access to treatment. Such investments will be essential to mitigate preventable mortality and disability from EMBC as global populations continue to age.

## Data Availability

The original contributions presented in the study are included in the article/**Supplementary Material**. Further inquiries can be directed to the corresponding author.
