# Supplementary material for: Global trends and disease burden of elderly male breast cancer, 1990-2021: a population-based study
Source: Front Endocrinol (Lausanne). 2026 Feb 6;17:1674679. doi: 10.3389/fendo.2026.1674679 (PMC12920204; doi:10.3389/fendo.2026.1674679)
Supplement: Supplementary file 1 [file Presentation1.pdf]

Supplementary Materials

Supplementary Methods 2

Figure S1. The relationship between the SDI and breast cancer mortality in older males across 204 countries and territories in 2021. 5

Figure S2. The joinpoint regression analysis of ASIR, ASMR, and ASDR across the five SDI regions. 6

Figure S3. AAPC in global and different SDI locations. 7

Figure S4. Global trends in ASIR, ASMR, and ASDR of breast cancer among older males from 1990 to 2021. 8

Figure S5. Global map of ASMR of EMBC in 1990 (A) and 2021 (B) and its AAPC (C). 9

Figure S6. Global map of ASDR of EMBC in 1990 (A) and 2021 (B) and its AAPC (C). 10

Table S1. The incident cases and age-standardized rates of breast cancer among women of reproductive age in 1990 and 2021, along with the AAPC from 1990 to 2021, across 204 countries and territories. 11

Table S2. The death cases and ASMR of older males with breast cancer in 1990 and 2021, and its AAPC from 1990 to 2021 20

Table S3. The DALYs and ASDR of older males with breast cancer in 1990 and 2021, and its AAPC from 1990 to 2021 31

Table S4. Changes in incident number according to population-level determinants and causes from 1990 to 2021. 41

Table S5. Changes in death number according to population-level determinants and causes from 1990 to 2021. 42

Table S6. Changes in DALYs number according to population-level determinants and causes from 1990 to 2021. 43

Supplementary Methods

All analyses in this study were performed using publicly accessible epidemiological estimates from the Global Burden of Disease (GBD) 2021 study compiled by the Institute for Health Metrics and Evaluation. This work represents secondary analysis of fully de-identified public data, requiring no ethical oversight. Data were extracted through the GHDx interface and validated using the EpiViz API. Extraction adhered to fixed parameters to ensure full reproducibility. Breast cancer was identified using ICD-10 code C50 (GBD cause ID 486), sex was restricted to male, and age groups comprised 60–64, 65–69, 70–74, 75–79, 80–84, 85–89, 90–94 and ⩾95 years. Annual counts and rates for incidence, mortality, and disability-adjusted life-years (DALYs), including crude and age-standardized metrics, were retrieved for each year between 1990 and 2021 across all GBD geographic levels. Age-standardized estimates were constructed using the GBD world standard population (2017 revision). Data were queried on 15 February 2024 via (https://ghdx.healthdata.org) and cross-validated with (https://vizhub.healthdata.org).

To ensure full reproducibility, the exact EpiViz API request structure used to retrieve EMBC estimates is reproduced below. This JSON payload is compatible with the EpiViz `/api/v1/estimates` endpoint and returns estimates for all locations, including the full set of 1,000 uncertainty draws used for uncertainty propagation.


```
{
  "cause_ids": [486],
  "measure_ids": [1, 2, 3],
  "metric_ids": [1, 2, 3],
  "age_group_ids": [15, 16, 17, 18, 19, 20, 30, 235],
  "sex_ids": [1],
  "location_ids": "all",
  "year_ids": [1990,1991,1992,1993,1994,1995,1996,1997,1998,1999,
            2000,2001,2002,2003,2004,2005,2006,2007,2008,2009,
            2010,2011,2012,2013,2014,2015,2016,2017,2018,2019,
            2020,2021],
  "include_aggregates": true,
```

"include_ihme_loc_ids": true,
"include_draws": true,
"num_draws": 1000
}

Analytical procedures were conducted using R (version 4.4.1, released June 2024), Stata (version 18.5, released April 2024), and the Joinpoint Regression Program (version 5.2.0, released June 2024). These versions accurately reflect the analytical timeframe of the study and ensure reproducibility of all outputs. Core R packages used included apc for age–period–cohort modelling, dplyr for data processing, ggplot2 for visualisation, ineq for inequality metrics, epitools for epidemiological computations, sf for spatial outputs, and boot for resampling-based uncertainty.

Temporal trend analysis employed log-linear Joinpoint regression. Up to five joinpoints were permitted, with a minimum segment length of three years. Model selection was based on the Monte-Carlo permutation method with 4,499 permutations and $\alpha = 0.05$. Poisson variance assumptions were assessed through deviance and Pearson dispersion diagnostics; scale corrections were applied when overdispersion was detected. Annual percent change estimates were derived for each segment, with confidence intervals calculated via the empirical quantile method. The average annual percent change was estimated by regressing $\ln(ASR_t)$ on calendar year and transforming the slope via $(\exp(b) - 1) \times 100\%$.

Age–period–cohort analyses followed established GBD workflows. Age groups comprised eight 5-year intervals beginning at 60–64 and ending at ⩾95 years. Periods were grouped into six intervals from 1990–1994 to 2017–2021; birth cohorts were defined as the difference between period midpoint and age midpoint. The intrinsic estimator method was used to resolve the identification problem inherent in APC modelling. Poisson regression with a log link was applied, and goodness-of-fit was evaluated using deviance residual patterns and dispersion statistics. To propagate uncertainty, APC models were fitted to all 1,000 GBD draws, and 95% uncertainty intervals were derived empirically. Sensitivity analyses included alternative elderly cutoffs (65+ and 75+), alternative reference categories, and alternative period boundaries.

Temporal decomposition of changes in incidence, mortality and DALYs from 1990 to 2021 used the Das Gupta standardization method. Total change was decomposed into contributions attributable to population growth, population ageing and age-specific rate changes, with interaction terms representing joint demographic–epidemiological influences. The decomposition identity

$$\Delta M = A + P + R + \tfrac{1}{2}(I_{PA} + I_{PR} + I_{AR}) + \tfrac{1}{4}I_{PAR}$$

was applied, where A represents ageing effects, P population growth, R rate effects and the I terms interaction components. To illustrate the approach, a multi-group worked example is provided. Suppose a population has four age groups, with 1990 mortality rates of 0.03, 0.05, 0.11 and 0.18 and 2021 mortality rates of 0.034, 0.058, 0.128 and 0.202, alongside population shifts from a younger-weighted to an older-weighted structure. Applying Das Gupta decomposition yields contributions of +21 deaths from population growth, +54 from ageing, +38 from rate changes and +12 from interaction terms, summing to the observed +125-death increase. This generalises the method to multi-group settings and aligns with epidemiological best practice for decomposition auditability.

Inequality analyses were performed using the slope index of inequality (SII) and concentration index (CI). SII was estimated via weighted least squares regression of each country's age-standardized outcome on its ridit-transformed SDI rank, with population as the analytic weight. CI was computed via the covariance of outcomes with fractional population ranks, scaled by the mean outcome, mathematically equivalent to twice the area between the Lorenz curve and the line of equality. Uncertainty intervals were obtained using 1,000-replicate bootstrap resampling. Fractional ranking was applied to handle ties.

Multiple testing was addressed using the Benjamini–Hochberg procedure with a threshold of $q < 0.05$. A sensitivity analysis using $q < 0.10$ produced comparable directional results, confirming the robustness of the primary inference.

All extraction scripts, data-processing pipelines, APC estimation loops and joining code used to generate figures and tables have been archived in a public repository.

Risk attributable burden estimates were obtained from the GBD 2021 comparative risk assessment framework. Attributable fractions for tobacco, dietary factors and alcohol use were estimated using exposure distributions, theoretical minimum risk exposure levels and relative risks sourced predominantly from long-term cohort studies of female breast cancer. As elderly men are substantially underrepresented in these studies and exhibit different hormonal environments, comorbidity profiles and baseline risks, transferability of risk estimates is limited. Potential misclassification, extrapolation from female cohorts and effect-modification biases should therefore be considered when interpreting these results.

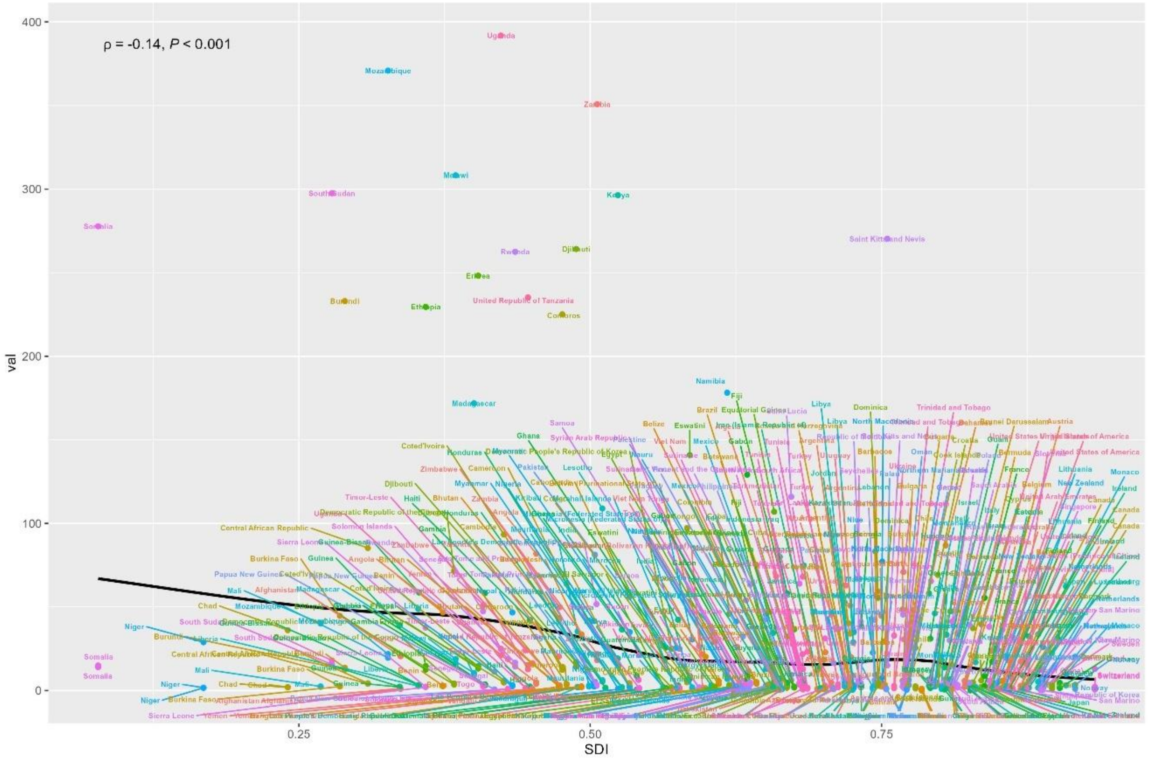

Figure S1. The relationship between the SDI and breast cancer mortality in older males across 204 countries and territories in 2021.

Abbreviations: SDI= Socio-demographic Index.

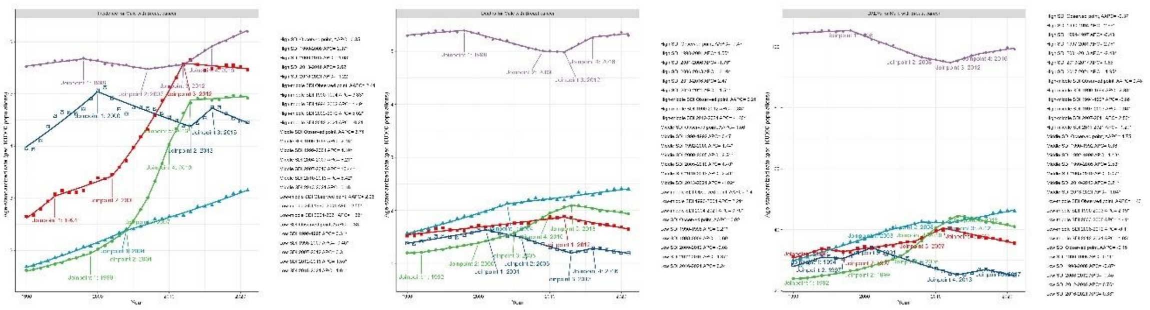

Figure S2. The joinpoint regression analysis of ASIR, ASMR, and ASDR across the five SDI regions.

Abbreviations: SDI: Sociodemographic Index, ASIR: age-standardised incidence rate, ASMR: age-standardised mortality rate, ASDR: age-standardised disability-adjusted life years rate.

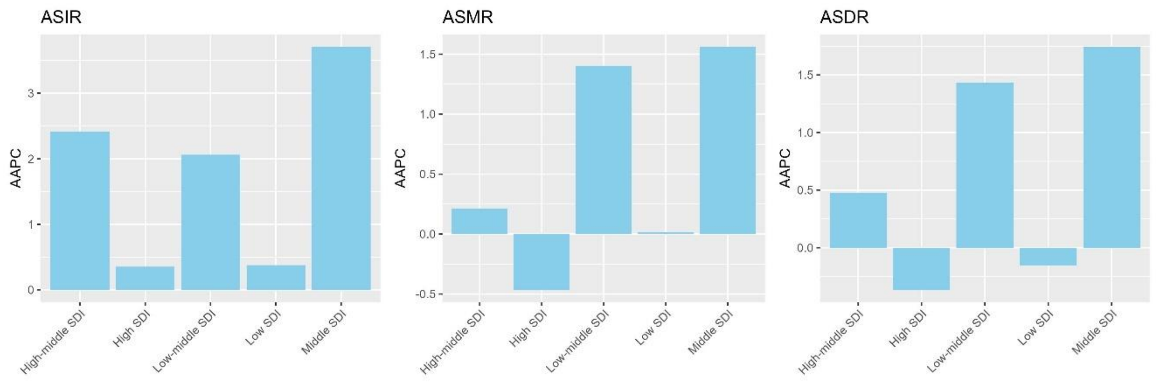

Figure S3. AAPC in global and different SDI locations.

Abbreviations: SDI: Sociodemographic Index, ASIR: age-standardised incidence rate, ASMR: age-standardised mortality rate, ASDR: age-standardised disability-adjusted life years rate.

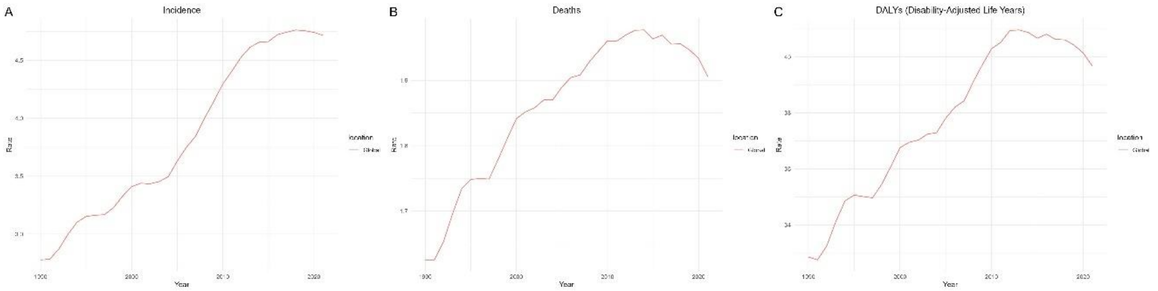

Figure S4. Global trends in ASIR, ASMR, and ASDR of breast cancer among older males from 1990 to 2021.

A: Trends of ASMR; B: Trends of ASIR; C: Trends of ASDR.

Abbreviations: SDI: Sociodemographic Index, ASIR: age-standardised incidence rate, ASMR: age-standardised mortality rate, ASDR: age-standardised disability-adjusted life years rate.

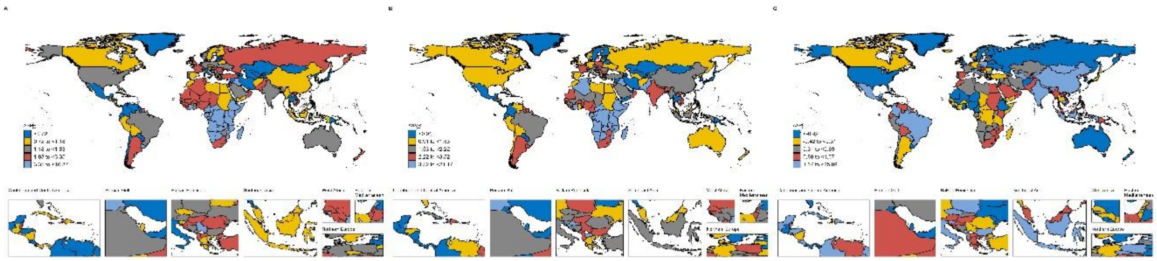

Figure S5. Global map of ASMR of EMBC in 1990 (A) and 2021 (B) and its AAPC (C).

Abbreviations: EMBC = Elderly male breast cancer; ASMR: age-standardised mortality rate; AAPC = average annual percentage change.

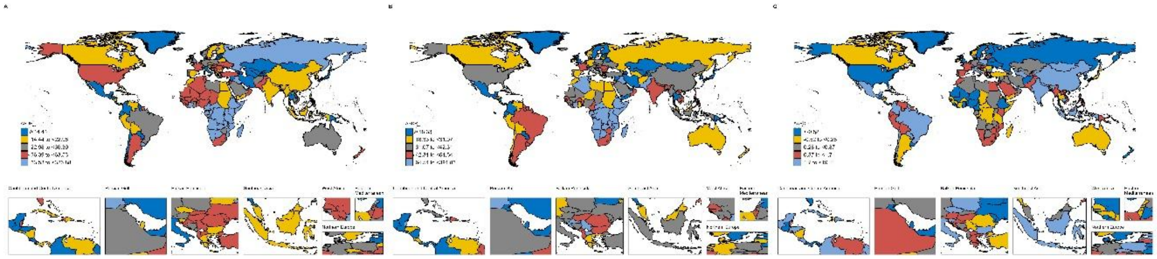

Figure S6. Global map of ASDR of EMBC in 1990 (A) and 2021 (B) and its AAPC (C).

Abbreviations: EMBC = Elderly male breast cancer; ASDR: age-standardised disability-adjusted life years rate; AAPC = average annual percentage change.

Table S1. The incident cases and age-standardized rates of breast cancer among women of reproductive age in 1990 and 2021, along with the AAPC from 1990 to 2021, across 204 countries and territories.

204 countries and territories	1990		2021		1990-2021
	Incident cases (95 % UI)	ASR per 100,000 (95 % UI)	Incident cases (95 % UI)	ASR per 100,000 (95 % UI)	AAPC (95 % CI)
Afghanistan	5.55（2.14-12.53）	1.2（0.46-2.72）	1.18（0.51-2.48）	1.18（0.51-2.48）	-0.06 (-0.16 to 0.04)
Albania	2.9（1.64-4.93）	3.01（1.71-5.13）	4.78（2.19-9.31）	4.78（2.19-9.31）	1.53 (1.01 to 2.06)
Algeria	21.77（11.43-37.55）	3.62（1.88-6.24）	6.44（2.74-12.53）	6.44（2.74-12.53）	1.87 (1.73 to 2)
American Samoa	0.01（0.01-0.02）	1.11（0.56-2.02）	1.42（0.76-2.54）	1.42（0.76-2.54）	0.92 (-0.16 to 2.03)
Andorra	0.1（0.05-0.2）	2.82（1.32-5.47）	2.84（1.2-5.68）	2.84（1.2-5.68）	0.03 (-0.93 to 1)
Angola	7.26（3.47-14.2）	3.8（1.78-7.46）	4.88（2.36-9.11）	4.88（2.36-9.11）	0.82 (0.6 to 1.04)
Antigua and Barbuda	0.06（0.04-0.07）	1.91（1.47-2.51）	4.01（2.82-5.67）	4.01（2.82-5.67）	2.24 (-0.97 to 5.55)
Argentina	64.71（44.09-94.36）	3.69（2.52-5.38）	4.69（3.11-6.82）	4.69（3.11-6.82）	1.03 (0.02 to 2.05)
Armenia	1.28（0.83-1.91）	1.03（0.67-1.53）	2.16（1.42-3.2）	2.16（1.42-3.2）	2 (-0.5 to 4.56)
Australia	44.82（29.03-66.75）	4.03（2.62-5.98）	4.62（2.84-6.83）	4.62（2.84-6.83）	0.73 (-0.7 to 2.18)
Austria	15.72（10.52-22.94）	2.76（1.85-4.01）	5.72（3.51-9.05）	5.72（3.51-9.05）	2.65 (1.54 to 3.77)
Azerbaijan	1.34（0.71-2.41）	0.69（0.36-1.24）	0.96（0.46-1.8）	0.96（0.46-1.8）	1.1 (0.82 to 1.38)
Bahamas	0.45（0.34-0.59）	6.12（4.63-8.07）	6.88（4.8-9.75）	6.88（4.8-9.75）	0.44 (-1.79 to 2.72)
Bahrain	0.16（0.08-0.28）	2.02（1.01-3.61）	3.24（1.46-6.43）	3.24（1.46-6.43）	1.55 (0.96 to 2.15)
Bangladesh	42.9（20.76-83.23）	1.47（0.71-2.85）	2.52（1.19-4.88）	2.52（1.19-4.88）	1.81 (1.1 to 2.52)
Barbados	0.77（0.58-1.02）	4.84（3.65-6.41）	5.4（3.6-7.88）	5.4（3.6-7.88）	0.53 (-0.76 to 1.84)
Belarus	8.02（5.21-12.04）	1.5（0.97-2.24）	0.12（0.07-0.19）	0.12（0.07-0.19）	-7.97 (-9.16 to -6.76)
Belgium	26.36（17.28-39.56）	3.24（2.13-4.83）	5.8（3.51-9.16）	5.8（3.51-9.16）	1.82 (0.84 to 2.8)
Belize	0.2（0.12-0.28）	3.59（2.08-5.08）	5.74（4.21-7.77）	5.74（4.21-7.77）	1.85 (-0.91 to 4.68)
Benin	3.27（1.64-6.01）	3.01（1.5-5.53）	2.92（1.35-5.87）	2.92（1.35-5.87）	-0.11 (-0.28 to 0.05)
Bermuda	0.07（0.04-0.11）	2.23（1.43-3.44）	5.77（3.4-	5.77（3.4-9.14）	2.99

			9.14）		(0.15 to 5.9)
Bhutan	0.18（0.08-0.36）	1.58（0.7-3.32）	3.11（1.3-6.21）	3.11（1.3-6.21）	2.26 (2.14 to 2.38)
Bolivia (Plurinational State of)	1.32（0.61-2.58）	0.81（0.37-1.58）	1.37（0.67-2.55）	1.37（0.67-2.55）	1.72 (1.51 to 1.94)
Bosnia and Herzegovina	3.92（1.84-7.71）	2.07（0.97-4.09）	3.5（1.69-6.37）	3.5（1.69-6.37）	1.68 (1.38 to 1.98)
Botswana	0.99（0.48-1.88）	4.04（1.94-7.71）	5.06（2.56-9.22）	5.06（2.56-9.22）	0.79 (0.41 to 1.18)
Brazil	72.78（64.09-81.85）	1.52（1.33-1.71）	3.76（3.25-4.35）	3.76（3.25-4.35）	3.01 (1.85 to 4.19)
Brunei Darussalam	0.1（0.05-0.18）	1.82（0.9-3.42）	1.35（0.67-2.65）	1.35（0.67-2.65）	-1.08 (-2.43 to 0.29)
Bulgaria	15.45（10.37-22.35）	2.18（1.47-3.12）	5.87（3.61-9.21）	5.87（3.61-9.21）	3.18 (2.05 to 4.32)
Burkina Faso	6.43（2.87-12.64）	2.82（1.25-5.59）	2.69（1.28-5.1）	2.69（1.28-5.1）	-0.16 (-0.37 to 0.06)
Burundi	17.72（8.76-32.88）	15.22（7.5-28.3）	13.61（6.41-26.46）	13.61（6.41-26.46）	-0.38 (-0.51 to -0.24)
Cabo Verde	0.25（0.1-0.66）	1.9（0.78-5.01）	4.45（1.9-9.01）	4.45（1.9-9.01）	2.73 (1.64 to 3.83)
Cambodia	2.64（1.27-4.97）	1.33（0.64-2.51）	2.88（1.19-5.43）	2.88（1.19-5.43）	2.52 (2.39 to 2.65)
Cameroon	9.5（4.74-17.55）	4.16（2.05-7.68）	4.01（1.71-8）	4.01（1.71-8）	-0.12 (-0.21 to -0.02)
Canada	49.75（32.23-74.49）	2.73（1.78-4.08）	3.37（2.03-5.21）	3.37（2.03-5.21）	1.21 (-0.14 to 2.58)
Central African Republic	2.49（1.11-5.11）	4.49（1.98-9.3）	4.38（1.97-8.91）	4.38（1.97-8.91）	-0.1 (-0.21 to 0.01)
Chad	2.95（1.24-6.31）	1.95（0.82-4.17）	2.06（0.91-4.3）	2.06（0.91-4.3）	0.17 (-0.03 to 0.36)
Chile	1.47（1.01-2.09）	0.27（0.19-0.39）	2.59（1.67-3.9）	2.59（1.67-3.9）	7.27 (4.16 to 10.47)
China	816.39（566.64-1185.31）	1.69（1.17-2.48）	6.81（2.81-10.03）	6.81（2.81-10.03）	4.6 (4.25 to 4.95)
Colombia	5.49（3.99-7.49）	0.57（0.41-0.77）	1.36（0.85-2.1）	1.36（0.85-2.1）	3.08 (1.57 to 4.61)
Comoros	1.21（0.48-2.34）	12.16（4.83-23.7）	14.18（4.49-31.13）	14.18（4.49-31.13）	0.5 (0.34 to 0.65)
Congo	2.93（1.49-5.15）	5.89（2.97-10.45）	6.03（2.96-11.3）	6.03（2.96-11.3）	0.07 (-0.05 to 0.2)
Cook Islands	0.01（0-0.02）	1.22（0.62-2.19）	2.5（1.12-4.78）	2.5（1.12-4.78）	2.34 (2.25 to 2.44)
Costa Rica	0.32（0.22-0.45）	0.32（0.22-0.46）	1.84（1.17-2.77）	1.84（1.17-2.77）	6.22 (5.21 to 7.24)
Coted'Ivoire	10.37（5.47-18.58）	5.01（2.65-8.94）	5.87（2.76-11.31）	5.87（2.76-11.31）	0.52 (0.42 to

					0.63)
Croatia	12.33（8.33-18.02）	4.6（3.14-6.67）	9.2（5.71-14.24）	9.2（5.71-14.24）	2.31 (1.44 to 3.2)
Cuba	7.1（4.69-10.43）	1.15（0.76-1.68）	3.35（2.08-5.18）	3.35（2.08-5.18）	3.53 (2.08 to 5.01)
Cyprus	2.17（1.08-3.95）	6.18（3.04-11.28）	6.04（2.88-11.28）	6.04（2.88-11.28）	-0.09 (-0.41 to 0.24)
Czechia	10.71（7.36-15.46）	1.51（1.05-2.17）	4.87（3.03-7.61）	4.87（3.03-7.61）	3.88 (2.83 to 4.94)
Democratic People's Republic of Korea	5.81（2.78-10.95）	0.9（0.43-1.69）	1.88（0.75-3.74）	1.88（0.75-3.74）	2.4 (2.26 to 2.55)
Democratic Republic of the Congo	29.02（13.04-57.84）	3.79（1.68-7.7）	4.19（1.77-9.11）	4.19（1.77-9.11）	0.32 (0.05 to 0.59)
Denmark	13.79（9.18-20.16）	3.06（2.04-4.46）	3.35（2.03-5.23）	3.35（2.03-5.23）	0.14 (-0.21 to 0.5)
Djibouti	0.7（0.32-1.35）	11.98（5.62-23.26）	16.94（6.89-35.29）	16.94（6.89-35.29）	1.11 (0.99 to 1.24)
Dominica	0.09（0.05-0.16）	2.96（1.51-5.4）	4.08（2.14-7.29）	4.08（2.14-7.29）	1.07 (0.81 to 1.32)
Dominican Republic	2.31（1.16-4.58）	1.14（0.57-2.29）	3.34（1.62-6.19）	3.34（1.62-6.19）	3.48 (2.95 to 4.02)
Ecuador	0.76（0.53-1.06）	0.26（0.18-0.37）	0.57（0.35-0.88）	0.57（0.35-0.88）	1.94 (-0.28 to 4.21)
Egypt	18.03（7.86-37.13）	1.23（0.54-2.57）	2.32（1.18-4.61）	2.32（1.18-4.61）	2.09 (1.71 to 2.47)
El Salvador	1.57（0.95-2.35）	0.96（0.59-1.44）	1.48（0.89-2.36）	1.48（0.89-2.36）	1.48 (0.31 to 2.66)
Equatorial Guinea	0.42（0.19-0.84）	4.56（2.09-9.17）	7.44（3.17-14.18）	7.44（3.17-14.18）	1.63 (1.43 to 1.82)
Eritrea	4.69（2.21-9.55）	12.37（5.74-25.01）	14.56（6.79-27.97）	14.56（6.79-27.97）	0.53 (0.42 to 0.63)
Estonia	0.76（0.5-1.12）	0.91（0.6-1.33）	2.6（1.56-4.15）	2.6（1.56-4.15）	3.58 (1.78 to 5.4)
Eswatini	0.75（0.39-1.32）	6.6（3.42-11.73）	8.7（4-16.14）	8.7（4-16.14）	0.95 (0.82 to 1.07)
Ethiopia	182.69（114.47-297.81）	16.58（10.32-27.28）	14.69（8.55-28.46）	14.69（8.55-28.46）	-0.39 (-0.45 to -0.33)
Fiji	0.3（0.14-0.56）	1.66（0.78-3.1）	2.37（1.07-4.38）	2.37（1.07-4.38）	1.11 (0.67 to 1.55)
Finland	6.55（4.4-9.57）	1.92（1.29-2.8）	1.41（0.85-2.23）	1.41（0.85-2.23）	-0.99 (-2.4 to 0.43)
France	187.37（133.25-260.96）	4.3（3.07-5.96）	8.71（5.3-13.69）	8.71（5.3-13.69）	2.54 (1.1 to 4.01)
Gabon	1.83（0.9-3.37）	6.16（3.03-11.42）	8.67（4.11-15.81）	8.67（4.11-15.81）	1.14 (1.01 to 1.26)
Gambia	0.56（0.27-1.05）	3.04（1.44-5.7）	3.72（1.74-7.27）	3.72（1.74-7.27）	0.63 (0.15 to

					1.12)
Georgia	0.01（0.01-0.01）	0（0-0）	3.29（2.42-4.47）	3.29（2.42-4.47）	25.46 (17.97 to 33.43)
Germany	141.57（97.43-202.46）	2.43（1.68-3.46）	4.66（2.99-7.09）	4.66（2.99-7.09）	2.13 (0.75 to 3.52)
Ghana	5.81（2.51-10.61）	1.74（0.77-3.16）	1.57（0.7-3.2）	1.57（0.7-3.2）	-0.29 (-0.51 to -0.06)
Greece	18.83（13.17-26.73）	2.1（1.47-2.97）	4.47（3-6.48）	4.47（3-6.48）	2.61 (1.88 to 3.34)
Greenland	0（0-0.01）	0.29（0.11-0.45）	0.28（0.11-0.47）	0.28（0.11-0.47）	-0.12 (-1.66 to 1.44)
Grenada	0.17（0.11-0.23）	4.33（2.82-5.85）	4.58（3.36-6.13）	4.58（3.36-6.13）	0.36 (-2.18 to 2.97)
Guam	0.01（0-0.04）	0.24（0.12-0.82）	1.15（0.45-1.84）	1.15（0.45-1.84）	5.11 (2.79 to 7.49)
Guatemala	1.28（0.97-1.67）	0.77（0.57-1）	0.6（0.44-0.81）	0.6（0.44-0.81）	-0.94 (-1.92 to 0.05)
Guinea	4.12（1.87-8.32）	2.16（0.97-4.35）	2.31（1.06-4.84）	2.31（1.06-4.84）	0.24 (0.15 to 0.33)
Guinea-Bissau	0.87（0.43-1.63）	4.06（1.98-7.62）	3.32（1.68-6.02）	3.32（1.68-6.02）	-0.66 (-0.78 to -0.53)
Guyana	0.01（0.01-0.01）	0.05（0.03-0.07）	2.92（1.84-4.46）	2.92（1.84-4.46）	14.05 (11.86 to 16.27)
Haiti	3.54（1.37-8.6）	2.08（0.8-5.02）	2.38（0.88-5.61）	2.38（0.88-5.61）	0.47 (0.29 to 0.64)
Honduras	0.82（0.37-1.6）	0.75（0.34-1.46）	1.73（0.87-3.22）	1.73（0.87-3.22）	2.79 (2.6 to 2.98)
Hungary	24.81（16.85-34.9）	3.22（2.2-4.52）	6.66（4.28-10.06）	6.66（4.28-10.06）	2.52 (2.14 to 2.9)
Iceland	0.59（0.38-0.88）	3.49（2.28-5.2）	5.64（3.37-8.87）	5.64（3.37-8.87）	1.6 (0.59 to 2.63)
India	306.86（223.01-425.28）	1.29（0.93-1.8）	3.07（1.66-4.01）	3.07（1.66-4.01）	2.9 (2.32 to 3.48)
Indonesia	44.06（27.53-62.35）	0.93（0.58-1.31）	2.42（1.02-3.62）	2.42（1.02-3.62）	3.12 (2.99 to 3.25)
Iran (Islamic Republic of)	11.66（7.33-20.05）	0.73（0.45-1.32）	1.47（0.89-2.55）	1.47（0.89-2.55）	2.21 (1.93 to 2.49)
Iraq	19.58（9.86-34.73）	4.57（2.3-8.12）	7.18（3.59-12.65）	7.18（3.59-12.65）	1.47 (1.27 to 1.68)
Ireland	5.09（3.36-7.46）	2.15（1.42-3.13）	3.12（1.87-4.95）	3.12（1.87-4.95）	1.29 (0.11 to 2.48)
Israel	13.44（8.73-19.98）	4.66（3.05-6.91）	6.69（4.08-10.44）	6.69（4.08-10.44）	1.2 (0.41 to 1.99)
Italy	49.71（36.47-67.84）	1.01（0.75-1.37）	5.59（3.8-7.82）	5.59（3.8-7.82）	5.77 (4.03 to 7.54)
Jamaica	4.71（3.04-6.93）	4.46（2.88-6.55）	6.28（3.8-9.85）	6.28（3.8-9.85）	1.21 (-0.01 to

					2.44)
Japan	94.98（69.36-130.34）	1.07（0.79-1.47）	1.5（1.03-2.09）	1.5（1.03-2.09）	0.89 (-0.18 to 1.98)
Jordan	1.87（0.92-3.42）	2.82（1.38-5.21）	3.52（1.66-6.8）	3.52（1.66-6.8）	0.77 (0.4 to 1.13)
Kazakhstan	2.59（1.6-3.86）	0.53（0.32-0.81）	0.79（0.51-1.17）	0.79（0.51-1.17）	1.35 (-0.03 to 2.75)
Kenya	45.96（29.86-89.56）	10.91（7.09-21.13）	19.15（10.19-35.78）	19.15（10.19-35.78）	1.83 (1.68 to 1.99)
Kiribati	0.03（0.02-0.05）	1.71（0.87-2.96）	2.03（0.98-3.7）	2.03（0.98-3.7）	0.56 (0.43 to 0.69)
Kuwait	0.76（0.5-1.14）	2.47（1.62-3.66）	2.25（1.32-3.65）	2.25（1.32-3.65）	0.71 (-0.89 to 2.34)
Kyrgyzstan	0.88（0.61-1.25）	0.74（0.52-1.04）	3.04（1.95-4.53）	3.04（1.95-4.53）	3.82 (0.18 to 7.6)
Lao People's Democratic Republic	1.43（0.63-2.82）	1.33（0.59-2.64）	2.23（0.96-4.19）	2.23（0.96-4.19）	1.68 (1.56 to 1.8)
Latvia	0.86（0.56-1.28）	0.58（0.38-0.86）	2.18（1.31-3.41）	2.18（1.31-3.41）	4.46 (3.24 to 5.68)
Lebanon	7.41（3.52-13.82）	6.42（3.06-11.84）	9.48（4.38-17.84）	9.48（4.38-17.84）	1.3 (1.02 to 1.58)
Lesotho	1.24（0.61-2.32）	3.89（1.9-7.33）	6.25（2.99-11.23）	6.25（2.99-11.23）	1.6 (1.33 to 1.86)
Liberia	2.12（1.01-4.01）	3.09（1.46-5.88）	3.13（1.23-7.11）	3.13（1.23-7.11）	0.01 (-0.24 to 0.26)
Libya	1.67（0.82-3.08）	1.52（0.74-2.82）	2.48（0.96-4.95）	2.48（0.96-4.95）	1.54 (0.8 to 2.28)
Lithuania	2.88（1.89-4.27）	1.41（0.93-2.08）	2.5（1.52-3.88）	2.5（1.52-3.88）	1.85 (0.88 to 2.83)
Luxembourg	0.74（0.54-1）	2.67（1.96-3.63）	5.28（3.46-7.87）	5.28（3.46-7.87）	2.18 (0.81 to 3.57)
Madagascar	26.62（13.07-50.89）	9.61（4.74-18.33）	10.43（4.8-20.43）	10.43（4.8-20.43）	0.25 (0 to 0.49)
Malawi	24.5（10.86-46.54）	13.36（5.95-25.58）	18.83（7.98-36.74）	18.83（7.98-36.74）	1.16 (0.99 to 1.33)
Malaysia	5.25（1.39-10.1）	1.07（0.28-2.07）	1.93（0.42-3.8）	1.93（0.42-3.8）	1.93 (1.62 to 2.23)
Maldives	0.06（0.03-0.11）	1.06（0.5-1.98）	1.22（0.59-2.23）	1.22（0.59-2.23）	0.35 (-0.01 to 0.71)
Mali	5.51（2.5-11.57）	2.67（1.2-5.57）	2.46（1.14-5.29）	2.46（1.14-5.29）	-0.23 (-0.36 to -0.1)
Malta	0.37（0.24-0.54）	1.56（1.03-2.27）	2.73（1.64-4.31）	2.73（1.64-4.31）	1.56 (0.22 to 2.93)
Marshall Islands	0.01（0-0.02）	0.87（0.36-1.98）	1.51（0.65-3.12）	1.51（0.65-3.12）	1.81 (1.63 to 1.99)
Mauritania	1.51（0.73-2.83）	3.08（1.47-5.77）	2.95（1-6.97）	2.95（1-6.97）	-0.11 (-0.23 to 0.01)

Mauritius	0.58（0.45-0.75）	1.58（1.23-2.03）	4.52（3.3-6.13）	4.52（3.3-6.13）	3.37 (-6.22 to 13.94)
Mexico	14.05（13.05-15.02）	0.61（0.57-0.66）	1.37（1.12-1.64）	1.37（1.12-1.64）	2.69 (1.06 to 4.34)
Micronesia (Federated States of)	0.03（0.01-0.05）	0.97（0.45-1.89）	1.89（0.87-3.5）	1.89（0.87-3.5）	2.16 (2.04 to 2.29)
Monaco	0.3（0.14-0.57）	7.56（3.57-14.16）	9.84（4.62-18.75）	9.84（4.62-18.75）	0.86 (0.77 to 0.96)
Mongolia	0.12（0.06-0.23）	0.23（0.11-0.46）	0.5（0.27-0.86）	0.5（0.27-0.86）	2.49 (1.96 to 3.03)
Montenegro	0.93（0.43-1.79）	2.94（1.36-5.64）	5.45（2.42-10.41）	5.45（2.42-10.41）	2.24 (1.4 to 3.09)
Morocco	8.99（4.3-16.45）	1.06（0.5-1.95）	2.02（0.98-3.75）	2.02（0.98-3.75）	2.12 (2.03 to 2.2)
Mozambique	38.15（18.88-71.71）	13.58（6.66-25.72）	21.81（10.75-40.37）	21.81（10.75-40.37）	1.56 (1.4 to 1.71)
Myanmar	14.13（6.69-26.23）	1.2（0.57-2.24）	2.33（0.99-4.29）	2.33（0.99-4.29）	2.15 (2.06 to 2.23)
Namibia	2.33（1.23-4.01）	7.73（4.01-13.46）	12.07（6.33-21.15）	12.07（6.33-21.15）	1.47 (1.38 to 1.57)
Nauru	0（0-0.01）	1.37（0.64-2.59）	2.56（1.16-4.9）	2.56（1.16-4.9）	2.04 (1.91 to 2.17)
Nepal	5.81（2.73-11.6）	1.22（0.57-2.44）	3.25（1.52-6.1）	3.25（1.52-6.1）	3.22 (3.08 to 3.36)
Netherlands	31.12（20.44-46.42）	2.94（1.94-4.38）	4.6（2.84-6.99）	4.6（2.84-6.99）	1.61 (1.09 to 2.14)
New Zealand	13.24（8.4-20.19）	5.91（3.77-8.98）	4.81（3-7.13）	4.81（3-7.13）	0.29 (-1.99 to 2.63)
Nicaragua	0.71（0.43-1.06）	0.93（0.56-1.39）	1.15（0.71-1.85）	1.15（0.71-1.85）	0.61 (-0.24 to 1.47)
Niger	3.14（1.41-6.7）	2.12（0.93-4.51）	1.72（0.68-3.91）	1.72（0.68-3.91）	-0.66 (-0.79 to -0.53)
Nigeria	64.05（35.43-103.92）	2.8（1.53-4.43）	2.74（1.43-5.13）	2.74（1.43-5.13）	-0.07 (-0.17 to 0.04)
Niue	0（0-0）	1.15（0.56-2.09）	2.62（1.25-4.8）	2.62（1.25-4.8）	2.69 (2.63 to 2.75)
North Macedonia	3.82（2.5-6.12）	3.75（2.46-6.01）	7.79（4.25-12.45）	7.79（4.25-12.45）	2.45 (1.92 to 2.98)
Northern Mariana Islands	0（0-0.01）	0.51（0.19-1.68）	0.85（0.37-2.26）	0.85（0.37-2.26）	1.65 (1.17 to 2.12)
Norway	9.52（7.06-12.92）	2.43（1.8-3.28）	3.39（2.23-4.84）	3.39（2.23-4.84）	1.53 (-1.06 to 4.18)
Oman	1.07（0.44-2.15）	3.16（1.26-6.37）	6.18（2.59-12.02）	6.18（2.59-12.02）	2.19 (1.03 to 3.36)
Pakistan	118.99（73.65-186.54）	3.36（2.06-5.3）	5.28（3.02-8.57）	5.28（3.02-8.57）	1.49 (1.4 to 1.58)

Palau	0.01（0-0.01）	1.1（0.52-2.06）	1.3（0.63-2.41）	1.3（0.63-2.41）	0.48 (0.34 to 0.62)
Palestine	1（0.49-1.86）	2.28（1.11-4.23）	2.9（1.48-5.29）	2.9（1.48-5.29）	0.81 (0.47 to 1.15)
Panama	1.26（0.96-1.66）	1.44（1.1-1.89）	1.51（0.99-2.18）	1.51（0.99-2.18）	0.1 (-0.53 to 0.73)
Papua New Guinea	0.41（0.14-0.99）	0.42（0.14-1.01）	0.66（0.21-1.63）	0.66（0.21-1.63）	1.52 (1.4 to 1.63)
Paraguay	0.48（0.24-0.83）	0.39（0.2-0.68）	0.92（0.44-1.69）	0.92（0.44-1.69）	2.86 (2.36 to 3.37)
Peru	6.25（3.24-11.03）	0.94（0.49-1.66）	1.82（0.89-3.3）	1.82（0.89-3.3）	2.34 (0.98 to 3.71)
Philippines	25.9（16.04-34.64）	1.81（1.12-2.41）	2.93（1.76-4.05）	2.93（1.76-4.05）	1.59 (1.36 to 1.82)
Poland	38.21（30.64-48.23）	1.81（1.46-2.26）	4.71（3.42-6.58）	4.71（3.42-6.58）	3 (0.77 to 5.29)
Portugal	20.51（14.27-28.88）	2.77（1.93-3.89）	6.1（3.76-9.43）	6.1（3.76-9.43）	2.61 (1.41 to 3.83)
Puerto Rico	3.04（2.01-4.4）	1.44（0.96-2.08）	4.56（2.8-7.08）	4.56（2.8-7.08）	3.96 (1 to 7.02)
Qatar	0.08（0.04-0.14）	1.62（0.76-3.1）	2.08（0.92-4.29）	2.08（0.92-4.29）	0.82 (-1.37 to 3.07)
Republic of Korea	14.05（7.26-23.78）	1.22（0.63-2.08）	1.05（0.53-1.93）	1.05（0.53-1.93）	-0.56 (-0.97 to -0.14)
Republic of Moldova	9.66（6.92-13.47）	4.7（3.39-6.5）	4.58（3.17-6.6）	4.58（3.17-6.6）	-0.18 (-1.67 to 1.34)
Romania	43.42（28.44-64.17）	2.9（1.91-4.26）	4.64（2.95-7.09）	4.64（2.95-7.09）	1.53 (0.85 to 2.22)
Russian Federation	411.02（379.05-448.23）	5.96（5.46-6.51）	3.91（3.32-4.52）	3.91（3.32-4.52）	-1.3 (-2.76 to 0.19)
Rwanda	22.16（10.2-40.7）	16.71（7.68-30.84）	16.65（6.78-32.84）	16.65（6.78-32.84）	-0.02 (-0.12 to 0.09)
Saint Kitts and Nevis	0.3（0.24-0.38）	14.86（11.5-18.81）	21.93（14.73-32.79）	21.93（14.73-32.79）	1.31 (-0.55 to 3.2)
Saint Lucia	0.17（0.13-0.22）	4.06（3.11-5.19）	9.41（6.61-13.17）	9.41（6.61-13.17）	3.05 (0.2 to 5.99)
Saint Vincent and the Grenadines	0.03（0.02-0.04）	0.8（0.63-1.02）	4.84（3.6-6.49）	4.84（3.6-6.49）	6.09 (3.3 to 8.96)
Samoa	0.06（0.03-0.1）	1.22（0.61-2.16）	1.84（0.9-3.37）	1.84（0.9-3.37）	1.33 (1.17 to 1.49)
San Marino	0.02（0.01-0.04）	0.96（0.47-1.71）	0.68（0.28-1.41）	0.68（0.28-1.41）	-1.26 (-1.81 to -0.71)
Sao Tome and Principe	0.1（0.05-0.2）	3.07（1.46-6.19）	3.62（1.58-7.35）	3.62（1.58-7.35）	0.55 (0.25 to 0.85)
Saudi Arabia	5.12（2.53-9.53）	1.6（0.79-2.96）	3.44（1.49-6.74）	3.44（1.49-6.74）	2.49 (2.37 to 2.6)
Senegal	5.36（2.55-10.25）	2.98（1.41-5.71）	3.04（1.34-	3.04（1.34-6.35）	0.03

			6.35）		(-0.16 to 0.22)
Serbia	28.18（14.7-49.11）	5.13（2.65-8.9）	7.3（3.66-13.24）	7.3（3.66-13.24）	1.26 (0.86 to 1.67)
Seychelles	0.14（0.07-0.26）	4.82（2.37-8.87）	5.65（2.81-10.2）	5.65（2.81-10.2）	0.47 (-0.6 to 1.56)
Sierra Leone	3.17（1.48-6.1）	2.66（1.24-5.15）	2.31（1.1-4.67）	2.31（1.1-4.67）	-0.46 (-0.56 to -0.36)
Singapore	0.99（0.67-1.41）	0.88（0.6-1.26）	0.98（0.6-1.52）	0.98（0.6-1.52）	0.16 (-1.06 to 1.4)
Slovakia	9.7（5.26-16.79）	3.04（1.65-5.24）	4.59（2.19-8.48）	4.59（2.19-8.48）	1.43 (1.05 to 1.82)
Slovenia	1.9（1.26-2.81）	1.64（1.09-2.41）	5.8（3.55-9.17）	5.8（3.55-9.17）	4.14 (3.21 to 5.07)
Solomon Islands	0.05（0.02-0.11）	0.58（0.2-1.34）	1.09（0.43-2.38）	1.09（0.43-2.38）	2.09 (1.88 to 2.31)
Somalia	13.79（6.13-26.84）	14.59（6.52-28.5）	14.99（6.68-31）	14.99（6.68-31）	0.11 (0.04 to 0.17)
South Africa	22.9（16.34-34.43）	2.57（1.83-3.83）	4.7（3.18-6.17）	4.7（3.18-6.17）	1.93 (1.6 to 2.27)
South Sudan	23.4（11.13-44.68）	13.59（6.46-26.07）	17.96（7.38-35.62）	17.96（7.38-35.62）	0.9 (0.82 to 0.99)
Spain	87.66（57.55-129.67）	2.86（1.88-4.22）	5.97（3.63-8.96）	5.97（3.63-8.96）	2.56 (2.11 to 3.02)
Sri Lanka	10.6（5.53-18.45）	1.82（0.94-3.18）	2.97（1.33-5.63）	2.97（1.33-5.63）	1.53 (0.5 to 2.58)
Sudan	5.09（2.07-11.13）	0.88（0.36-1.93）	1.48（0.71-2.73）	1.48（0.71-2.73）	1.69 (1.63 to 1.74)
Suriname	0.33（0.17-0.56）	2.31（1.24-4）	3.71（1.76-6.86）	3.71（1.76-6.86）	1.65 (0.8 to 2.5)
Sweden	16.91（11.56-24.01）	1.93（1.32-2.73）	2.01（1.26-3.07）	2.01（1.26-3.07）	0.49 (-0.57 to 1.56)
Switzerland	16.41（10.53-24.4）	2.99（1.92-4.44）	2.18（1.27-3.49）	2.18（1.27-3.49）	-1.09 (-1.94 to -0.24)
Syrian Arab Republic	0.41（0.2-0.76）	0.13（0.06-0.25）	0.26（0.12-0.5）	0.26（0.12-0.5）	2.16 (1.88 to 2.45)
Taiwan (Province of China)	8.2（5.67-11.79）	0.74（0.52-1.06）	2.06（1.28-3.2）	2.06（1.28-3.2）	2.95 (1.89 to 4.03)
Tajikistan	0.36（0.15-0.79）	0.28（0.12-0.63）	0.44（0.18-0.95）	0.44（0.18-0.95）	1.31 (0.78 to 1.84)
Thailand	13.98（7.35-24.34）	0.83（0.44-1.46）	1.26（0.6-2.25）	1.26（0.6-2.25）	1.34 (0.97 to 1.71)
Timor-Leste	0.09（0.04-0.17）	0.75（0.35-1.42）	1.56（0.65-2.96）	1.56（0.65-2.96）	2.38 (2.26 to 2.5)
Togo	1.66（0.78-3.39）	2.79（1.3-5.68）	3.2（1.54-6.19）	3.2（1.54-6.19）	0.44 (0.33 to 0.56)
Tokelau	0（0-0）	0.77（0.33-1.58）	1.69（0.8-3.22）	1.69（0.8-3.22）	2.54 (2.42 to 2.67)

Tonga	0.03（0.01-0.05）	0.83（0.39-1.63）	1.88（0.86-3.53）	1.88（0.86-3.53）	2.66 (2.22 to 3.1)
Trinidad and Tobago	2.8（2.18-3.58）	6.16（4.81-7.83）	5.68（3.76-8.38）	5.68（3.76-8.38）	-0.14 (-2.06 to 1.82)
Tunisia	11.34（5.58-20.79）	3.9（1.92-7.13）	6.97（2.87-13.94）	6.97（2.87-13.94）	1.86 (1.68 to 2.04)
Turkey	43.63（21.5-81.44）	2.48（1.21-4.65）	3.75（1.88-6.84）	3.75（1.88-6.84）	1.3 (1.07 to 1.53)
Turkmenistan	0.65（0.43-0.93）	0.82（0.53-1.17）	1.63（0.97-2.64）	1.63（0.97-2.64）	2.24 (1.94 to 2.54)
Tuvalu	0（0-0.01）	0.77（0.31-1.71）	1.6（0.73-3.06）	1.6（0.73-3.06）	2.39 (2.31 to 2.47)
Uganda	58.73（31.19-101.06）	17.85（9.43-30.75）	24.98（12.48-45.27）	24.98（12.48-45.27）	1.09 (0.94 to 1.24)
Ukraine	90.72（60.42-132.85）	2.8（1.86-4.09）	2.72（1.56-4.53）	2.72（1.56-4.53）	-0.01 (-0.77 to 0.75)
United Arab Emirates	0.17（0.07-0.39）	0.9（0.38-2.04）	1.03（0.51-1.9）	1.03（0.51-1.9）	0.11 (-0.79 to 1.03)
United Kingdom	169.51（153.22-187.48）	3.46（3.12-3.82）	2.61（2.27-2.96）	2.61（2.27-2.96）	-1.01 (-1.66 to -0.35)
United Republic of Tanzania	79.85（38.78-150.44）	14.11（6.83-26.7）	14.97（6.59-29.99）	14.97（6.59-29.99）	0.19 (0.1 to 0.29)
United States of America	1389.45（1272.12-1505.72）	7.82（7.14-8.48）	6.93（6.31-7.46）	6.93（6.31-7.46）	1.72 (0.89 to 2.56)
United States Virgin Islands	0.14（0.07-0.27）	3.38（1.65-6.65）	5.56（2.71-10.37）	5.56（2.71-10.37）	-0.29 (-1.05 to 0.48)
Uruguay	13.87（9.42-19.95）	6.33（4.3-9.1）	6.28（4.08-9.39）	6.28（4.08-9.39）	-0.27 (-1.65 to 1.12)
Uzbekistan	1.48（0.73-2.34）	0.3（0.14-0.48）	0.42（0.28-0.62）	0.42（0.28-0.62）	1.19 (-0.89 to 3.31)
Vanuatu	0.02（0.01-0.05）	0.62（0.23-1.4）	1.19（0.5-2.52）	1.19（0.5-2.52）	2.11 (1.99 to 2.24)
Venezuela (Bolivarian Republic of)	4.61（3.58-5.94）	0.9（0.7-1.15）	1.73（1.12-2.56）	1.73（1.12-2.56）	2.26 (0.44 to 4.11)
Viet Nam	41.59（21.67-71.44）	2.23（1.16-3.84）	5.06（2.45-9.16）	5.06（2.45-9.16）	2.69 (2.53 to 2.85)
Yemen	2.41（0.9-5.44）	0.92（0.34-2.08）	1.05（0.42-2.28）	1.05（0.42-2.28）	0.43 (0.23 to 0.63)
Zambia	22.96（11.29-42.29）	14.85（7.24-27.55）	21.79（8.1-52.84）	21.79（8.1-52.84）	1.3 (1.07 to 1.54)
Zimbabwe	3.65（1.88-6.5）	1.92（0.99-3.41）	3.08（1.57-5.51）	3.08（1.57-5.51）	1.61 (1.36 to 1.85)

Abbreviations: SDI: Sociodemographic Index; ASIR: age-standardised incidence rate; AAPC=average annual percentage change; CI = confidence interval; UI = uncertainty interval.

Table S2. The death cases and ASMR of older males with breast cancer in 1990 and 2021, and its AAPC from 1990 to 2021

Characteristics	1990		2021		1990-2021
	Death cases (95 % UI)	ASMR (95 % UI)	Death cases (95 % UI)	ASMR (95 % UI)	AAPC (95 % CI)
Global	3159.55（2712.83-3845.56）	1.63（1.39-1.97）	9083.73（6374.31-11060.48）	1.91（1.35-2.31）	0.58 (0.38 to 0.77)
High SDI	764.67（715.5-806）	1.52（1.34-1.74）	1509.66（1353.76-1637.37）	1.65（1.16-2.01）	-0.47 (-0.99 to 0.06)
High-middle SDI	707.05（623.11-806.88）	1.57（1.19-2.23）	1777.24（1223.67-2182.73）	2.4（1.67-3.11）	0.21 (0.07 to 0.36)
Low SDI	607.71（446.36-901.66）	5.29（3.88-7.93）	1234（903.17-1959.04）	5.3（3.88-8.34）	0.02 (-0.13 to 0.16)
Low-middle SDI	478.03（366.79-671.2）	1.38（1.28-1.46）	1734.81（1209.82-2239.68）	1.19（1.07-1.29）	1.4 (1.31 to 1.49)
Middle SDI	598.49（439.05-	1.2（0.88-	2818.55（1431.36-	1.94（1.01-	1.56

	758.9）	1.51）	3665.99）	2.5）	(1.35 to 1.78)
Region					
Andean Latin America	7.02（4.27-11.37）	0.64（0.39-1.04）	27.22（16.61-42.2）	0.82（0.5-1.27）	0.9 (0 to 1.81)
Australasia	18.8（15-23.15）	1.54（1.23-1.9）	40.51（30.73-51.74）	1.2（0.91-1.53）	-0.61 (-2.61 to 1.42)
Caribbean	20.24（16.3-26.05）	1.41（1.14-1.81）	67.51（52.92-85.04）	2.2（1.72-2.77）	1.44 (0.01 to 2.89)
Central Asia	6.11（4.55-7.64）	0.33（0.24-0.42）	23.72（20.33-27.56）	0.7（0.6-0.81）	2.01 (0.01 to 4.05)
Central Europe	118.09（101.7-137.17）	1.68（1.44-1.95）	288.92（248.93-329.33）	2.41（2.07-2.74）	1.27 (0.58 to 1.96)
Central Latin America	23.41（21.43-25.38）	0.54（0.49-0.59）	111.85（95.95-128.84）	0.82（0.7-0.95）	1.45 (0.47 to 2.44)
Central Sub-Saharan Africa	39.01（21.41-69.64）	3.8（2.05-6.98）	83.37（46.8-153.66）	3.89（2.15-7.38）	0.08 (-0.06 to 0.22)
East Asia	444.23（311.67-634.39）	0.97（0.68-1.41）	2176.66（900.27-3141.22）	1.69（0.73-2.41）	1.78 (1.51 to 2.06)
Eastern Europe	263.59（238.95-288.74）	2.48（2.24-2.71）	219.72（185.02-256.55）	1.31（1.1-1.52）	-1.98 (-3.14 to -0.8)
Eastern Sub-Saharan Africa	510.66（372.9-807.23）	13.97（10.15-22.4）	1040.26（674.75-1841.84）	14.41（9.41-25.39）	0.11 (0.08 to 0.14)
High-income Asia Pacific	49.53（44.26-56.05）	0.53（0.47-0.6）	125.09（109.14-140.14）	0.42（0.37-0.48）	-0.69 (-1.14 to -0.24)
High-income North America	312.89（294.38-328.51）	1.66（1.55-1.75）	540.16（492.68-574.98）	1.36（1.24-1.45）	-0.54 (-1.35 to 0.28)
North Africa and Middle East	128.66（87.54-191.37）	1.5（1.02-2.23）	404.77（282.05-579.73）	1.75（1.21-2.51）	0.49 (0.38 to 0.6)
Oceania	0.83（0.43-1.5）	0.57（0.3-1.01）	2.87（1.41-5.4）	0.75（0.37-1.39）	0.91 (0.62 to 1.2)
South Asia	417.91（309.93-589.28）	1.48（1.08-2.1）	1794.11（1070.33-2325.9）	2.42（1.45-3.14）	1.69 (1.07 to 2.32)
Southeast Asia	129.89（84.24-168.23）	1.13（0.73-1.47）	536.42（279.94-699.49）	1.71（0.89-2.23）	1.35 (1.26 to 1.43)
Southern Latin America	55.95（42.27-73.1）	2.34（1.76-3.07）	102.97（80.15-128.75）	2.17（1.69-2.72）	-0.12 (-2.45 to 2.26)
Southern Sub-Saharan Africa	26.54（19.43-38.07）	2.43（1.76-3.45）	80.28（55.23-101.29）	3.64（2.48-4.57）	1.32 (1.09 to 1.55)
Tropical Latin America	54.8（48.81-61.17）	1.17（1.03-1.31）	299.73（263.34-335.44）	2.14（1.88-2.4）	2.03 (0.74 to 3.34)
Western Europe	415.55（378.37-451.68）	1.44（1.3-1.57）	910.57（779.54-1028.76）	1.6（1.37-1.8）	0.46 (-0.47 to 1.4)
Western Sub-Saharan Africa	115.85（66.26-175.91）	2.7（1.52-4.05）	207.04（130.41-357.38）	2.47（1.51-4.16）	-0.28 (-0.34 to -0.22)
204 countries and territories					
Afghanistan	4.99（1.92-11.27）	1.12（0.43-2.55）	3.45（1.49-7.27）	0.96（0.41-2.04）	-0.49 (-0.55 to -0.43)

Albania	2.01（1.18-3.26）	2.33（1.36-3.82）	6.53（3.16-12.46）	2.6（1.27-4.95）	0.31 (-0.06 to 0.68)
Algeria	17.16（9.23-29.18）	3.19（1.69-5.41）	70.72（31.09-130.74）	4.08（1.77-7.55）	0.83 (0.69 to 0.97)
American Samoa	0.01（0-0.02）	0.86（0.44-1.52）	0.03（0.01-0.04）	0.96（0.53-1.66）	0.48 (-0.57 to 1.54)
Andorra	0.04（0.02-0.08）	1.36（0.67-2.48）	0.09（0.04-0.16）	0.9（0.43-1.67）	-1.41 (-2.23 to -0.58)
Angola	6.52（3.11-12.8）	3.65（1.7-7.17）	18.84（9.21-34.49）	4.12（1.98-7.65）	0.41 (0.21 to 0.61)
Antigua and Barbuda	0.04（0.03-0.05）	1.4（1.15-1.69）	0.13（0.1-0.17）	2.38（1.88-3.04）	1.71 (-1.42 to 4.95)
Argentina	45.83（32.77-63.16）	2.75（1.96-3.79）	77.32（56.8-102.17）	2.56（1.88-3.39）	0.08 (-0.92 to 1.08)
Armenia	0.81（0.55-1.12）	0.7（0.48-0.98）	2.62（1.89-3.55）	1.15（0.83-1.56）	1.16 (-1.38 to 3.77)
Australia	14.42（11.03-18.39）	1.42（1.08-1.82）	33.66（24.57-44.58）	1.18（0.86-1.56）	-0.45 (-1.91 to 1.04)
Austria	7.81（5.87-10.19）	1.43（1.07-1.86）	19.68（13.99-26.55）	1.84（1.31-2.48）	0.97 (0.11 to 1.84)
Azerbaijan	0.95（0.5-1.65）	0.55（0.28-0.96）	2.6（1.33-4.66）	0.6（0.31-1.08）	0.32 (0.03 to 0.62)
Bahamas	0.32（0.26-0.39）	4.6（3.7-5.62）	0.86（0.65-1.1）	4.35（3.3-5.59）	-0.16 (-2.39 to 2.13)
Bahrain	0.11（0.06-0.19）	1.55（0.8-2.71）	0.53（0.25-1.05）	1.64（0.79-3.1）	0.19 (-0.61 to 1)
Bangladesh	39.07（18.89-76.3）	1.37（0.66-2.68）	146.96（70.92-289.48）	1.89（0.92-3.68）	1.18 (0.48 to 1.89)
Barbados	0.56（0.45-0.68）	3.58（2.92-4.36）	0.95（0.7-1.26）	3.19（2.36-4.23）	-0.21 (-1.4 to 0.99)
Belarus	4.05（2.91-5.44）	0.8（0.57-1.08）	0.29（0.2-0.41）	0.04（0.03-0.05）	-9.49 (-10.8 to -8.15)
Belgium	12.98（9.52-17.24）	1.69（1.24-2.25）	26.58（18.83-35.94）	1.88（1.33-2.54）	0.31 (-0.4 to 1.02)
Belize	0.16（0.09-0.21）	2.87（1.73-3.89）	0.63（0.5-0.79）	3.84（3.03-4.78）	1.3 (-1.38 to 4.06)
Benin	3（1.51-5.45）	2.87（1.44-5.2）	5.48（2.54-11.13）	2.57（1.19-5.12）	-0.36 (-0.49 to -0.22)
Bermuda	0.04（0.03-0.06）	1.47（1.01-2.16）	0.17（0.12-0.25）	2.23（1.53-3.19）	1.18 (-1.03 to 3.44)
Bhutan	0.15（0.07-0.32）	1.52（0.67-3.23）	0.81（0.34-1.59）	2.41（1.03-4.73）	1.51 (1.41 to 1.62)
Bolivia (Plurinational State of)	1.19（0.56-2.38）	0.76（0.36-1.52）	4.88（2.39-8.85）	1.03（0.51-1.86）	0.98 (0.78 to 1.17)
Bosnia and Herzegovina	2.4（1.14-4.59）	1.36（0.64-2.62）	5.95（3.04-10.2）	1.7（0.87-2.9）	0.76 (0.34 to 1.18)
Botswana	0.83（0.4-1.53）	3.76（1.79-	2.18（1.11-3.89）	4.11（2.1-	0.37

		6.97）		7.32）	(0.13 to 0.6)
Brazil	54.44（48.51-60.84）	1.19（1.05-1.34）	297.94（261.79-333.52）	2.18（1.91-2.45）	2.02 (0.72 to 3.33)
Brunei Darussalam	0.07（0.04-0.13）	1.41（0.72-2.61）	0.11（0.06-0.21）	0.87（0.46-1.64）	-1.69 (-2.84 to -0.52)
Bulgaria	7.25（5.43-9.36）	1.18（0.88-1.53）	18.47（12.77-25.94）	2.43（1.69-3.4）	2.27 (1.09 to 3.47)
Burkina Faso	5.65（2.5-11.1）	2.67（1.16-5.28）	9.69（4.64-18.66）	2.4（1.15-4.6）	-0.32 (-0.52 to -0.13)
Burundi	16.49（8.2-30.49）	14.78（7.31-27.42）	28.12（13.28-55.27）	12.51（5.91-24.43）	-0.57 (-0.68 to -0.46)
Cabo Verde	0.21（0.09-0.55）	1.65（0.69-4.18）	0.61（0.27-1.2）	3.07（1.38-6.01）	1.95 (0.69 to 3.24)
Cambodia	2.36（1.13-4.37）	1.27（0.6-2.36）	10.86（4.54-20.15）	2.24（0.95-4.17）	1.86 (1.78 to 1.93)
Cameroon	8.29（4.1-15.14）	3.9（1.9-7.14）	18.1（7.92-35.34）	3.4（1.46-6.63）	-0.43 (-0.52 to -0.34)
Canada	19.11（14.36-24.67）	1.11（0.83-1.44）	42.11（30.6-56.52）	0.95（0.69-1.27）	-0.13 (-1.36 to 1.11)
Central African Republic	2.25（1.02-4.65）	4.34（1.94-9.09）	3.41（1.57-7.01）	4.08（1.81-8.32）	-0.22 (-0.3 to -0.15)
Chad	2.72（1.13-5.79）	1.88（0.78-4.02）	5.59（2.51-11.32）	1.89（0.84-3.84）	0 (-0.18 to 0.18)
Chile	0.98（0.72-1.32）	0.19（0.14-0.25）	16.08（11.68-21.68）	1.13（0.82-1.53）	5.56 (2.38 to 8.84)
China	435.93（304.53-625）	0.99（0.69-1.45）	2141.81（874.54-3104.51）	1.72（0.74-2.47）	1.79 (1.51 to 2.06)
Colombia	4.11（3.11-5.36）	0.44（0.33-0.58）	21.79（15.11-30.3）	0.71（0.49-0.99）	1.91 (0.87 to 2.95)
Comoros	1.07（0.42-2.07）	11.61（4.59-22.72）	2.66（0.85-5.89）	12.4（3.96-27.32）	0.18 (0.09 to 0.27)
Congo	2.58（1.32-4.52）	5.54（2.79-9.83）	5.51（2.8-9.86）	4.94（2.49-9.05）	-0.35 (-0.43 to -0.27)
Cook Islands	0.01（0-0.01）	0.89（0.46-1.52）	0.02（0.01-0.03）	1.22（0.58-2.2）	1.06 (0.96 to 1.17)
Costa Rica	0.22（0.16-0.29）	0.23（0.16-0.3）	2.78（1.99-3.82）	0.88（0.63-1.22）	4.83 (3.85 to 5.81)
Coted'Ivoire	8.82（4.66-15.67）	4.65（2.45-8.28）	24.77（11.78-47.95）	4.8（2.28-9.15）	0.09 (0 to 0.18)
Croatia	6.09（4.6-7.92）	2.53（1.9-3.3）	17.68（12.92-23.88）	3.59（2.62-4.84）	1.22 (0.36 to 2.09)
Cuba	4.82（3.38-6.66）	0.81（0.57-1.12）	18.79（12.81-26.38）	1.64（1.12-2.3）	2.61 (1.22 to 4.02)
Cyprus	1.3（0.68-2.27）	4.38（2.22-7.77）	2.44（1.31-4.22）	2.18（1.16-3.76）	-2.24 (-2.55 to -1.93)
Czechia	5.7（4.33-7.3）	0.86（0.66-1.11）	22.93（16.46-31.03）	1.91（1.37-2.59）	2.73 (1.78 to 3.69)

Democratic People's Republic of Korea	4.13（2.01-7.64）	0.68（0.33-1.27）	16.81（6.71-32.44）	1.09（0.44-2.12）	1.51 (1.35 to 1.68)
Democratic Republic of the Congo	25.7（11.67-51.37）	3.6（1.6-7.39）	51.47（22.35-108.73）	3.59（1.53-7.81）	-0.01 (-0.16 to 0.15)
Denmark	7.43（5.44-9.84）	1.7（1.25-2.26）	8.39（5.92-11.49）	1.14（0.81-1.57）	-1.54 (-2.02 to -1.06)
Djibouti	0.6（0.28-1.19）	11.33（5.35-22.34）	3.89（1.62-7.99）	14.36（6.05-29.29）	0.77 (0.72 to 0.82)
Dominica	0.07（0.04-0.12）	2.41（1.25-4.31）	0.13（0.07-0.22）	2.94（1.58-5.05）	0.69 (0.58 to 0.79)
Dominican Republic	1.92（0.98-3.77）	1（0.51-1.98）	13.08（6.57-23.57）	2.33（1.17-4.21）	2.73 (2.24 to 3.23)
Ecuador	0.64（0.46-0.86）	0.23（0.17-0.31）	3.22（2.07-4.79）	0.37（0.24-0.54）	0.91 (-1.26 to 3.14)
Egypt	14.35（6.44-29.39）	1.05（0.47-2.17）	48.66（26.36-93.79）	1.41（0.75-2.76）	0.98 (0.57 to 1.39)
El Salvador	1.29（0.81-1.89）	0.8（0.5-1.17）	2.8（1.82-4.17）	0.85（0.55-1.26）	0.32 (-0.42 to 1.06)
Equatorial Guinea	0.38（0.18-0.76）	4.38（2.01-8.82）	1.03（0.46-1.94）	5.47（2.43-10.31）	0.74 (0.59 to 0.89)
Eritrea	4.14（1.97-8.32）	11.97（5.61-24.1）	11.04（5.2-21.47）	13.19（6.19-25.64）	0.31 (0.22 to 0.4)
Estonia	0.37（0.27-0.48）	0.47（0.35-0.62）	1.07（0.75-1.47）	0.84（0.58-1.15）	1.85 (-0.11 to 3.86)
Eswatini	0.63（0.33-1.1）	6.08（3.18-10.71）	1.37（0.64-2.49）	7.05（3.23-13.04）	0.53 (0.38 to 0.68)
Ethiopia	169.26（106.22-276.49）	16.42（10.22-27.2）	271.2（162.07-528.13）	12.54（7.53-24.11）	-0.88 (-0.97 to -0.79)
Fiji	0.23（0.11-0.43）	1.37（0.64-2.53）	0.66（0.3-1.21）	1.77（0.81-3.24）	0.83 (0.09 to 1.57)
Finland	3.44（2.52-4.54）	1.08（0.79-1.43）	3.42（2.41-4.77）	0.44（0.31-0.61）	-2.86 (-4.48 to -1.22)
France	99.68（78.83-120.78）	2.39（1.89-2.91）	231.8（164.38-317.78）	2.74（1.94-3.75）	0.67 (-0.81 to 2.18)
Gabon	1.58（0.78-2.88）	5.6（2.77-10.31）	3.11（1.55-5.52）	6.61（3.26-11.89）	0.58 (0.43 to 0.74)
Gambia	0.47（0.22-0.89）	2.79（1.3-5.29）	1.35（0.66-2.6）	3.13（1.5-6.03）	0.36 (-0.03 to 0.76)
Georgia	0（0-0.01）	0（0-0）	5.7（4.69-6.85）	1.94（1.6-2.33）	25.09 (17.55 to 33.11)
Germany	79.03（59.26-103.58）	1.42（1.07-1.86）	175.39（132.16-225.15）	1.48（1.12-1.9）	0.27 (-0.89 to 1.44)
Ghana	4.77（2.13-8.73）	1.52（0.7-2.78）	8.97（4.09-17.89）	1.19（0.53-2.5）	-0.74 (-0.99 to -0.5)
Greece	8.41（6.99-10.01）	0.97（0.81-1.16）	23.43（19.13-28.19）	1.51（1.24-1.82）	1.56 (0.74 to 2.39)
Greenland	0（0-0）	0.2（0.08-	0.01（0-0.01）	0.14（0.06-	-1.06

		0.31）		0.22）	(-2.72 to 0.63)
Grenada	0.14（0.09-0.18）	3.58（2.37-4.66）	0.16（0.13-0.19）	3.14（2.5-3.85）	-0.37 (-2.78 to 2.11)
Guam	0.01（0-0.02）	0.16（0.08-0.52）	0.08（0.03-0.11）	0.62（0.25-0.9）	4.35 (1.99 to 6.76)
Guatemala	1.13（0.87-1.45）	0.76（0.56-0.97）	2.53（1.97-3.18）	0.44（0.34-0.55）	-1.86 (-2.79 to -0.92)
Guinea	3.82（1.73-7.73）	2.1（0.94-4.24）	5.99（2.8-12.84）	2.09（0.98-4.43）	0 (-0.1 to 0.11)
Guinea-Bissau	0.79（0.38-1.46）	3.92（1.88-7.29）	0.84（0.44-1.5）	3.02（1.53-5.43）	-0.85 (-0.91 to -0.79)
Guyana	0.01（0.01-0.01）	0.04（0.03-0.06）	0.74（0.48-1.09）	2.24（1.45-3.31）	13.44 (11.3 to 15.62)
Haiti	3.26（1.27-7.89）	2.04（0.78-4.88）	7.23（2.7-16.93）	2.13（0.79-4.98）	0.16 (-0.01 to 0.34)
Honduras	0.7（0.33-1.37）	0.67（0.31-1.32）	4.23（2.14-7.69）	1.3（0.66-2.36）	2.15 (2.03 to 2.26)
Hungary	14.23（10.56-18.34）	1.97（1.46-2.55）	28.4（20.8-37.39）	2.8（2.04-3.69）	1.07 (0.26 to 1.9)
Iceland	0.27（0.2-0.35）	1.6（1.19-2.09）	0.6（0.43-0.8）	1.62（1.15-2.16）	0.2 (-0.57 to 0.96)
India	264.41（191.57-368.31）	1.19（0.86-1.67）	1353.11（734.39-1764.33）	2.29（1.24-2.98）	2.15 (1.44 to 2.86)
Indonesia	37.13（23.32-52.67）	0.84（0.53-1.18）	195.91（83.64-290.84）	1.82（0.8-2.69）	2.54 (2.45 to 2.63)
Iran (Islamic Republic of)	7.75（5.18-13.49）	0.53（0.34-0.99）	29.44（19.97-49.76）	0.68（0.46-1.18）	0.79 (0.52 to 1.07)
Iraq	15.14（7.82-26.51）	3.6（1.86-6.3）	43.86（22.48-73.67）	4.12（2.11-6.95）	0.45 (0.05 to 0.85)
Ireland	2.56（1.9-3.36）	1.15（0.85-1.51）	4.59（3.18-6.31）	0.96（0.66-1.31）	-0.38 (-1.53 to 0.79)
Israel	7.66（5.51-10.46）	2.74（1.98-3.73）	17.95（12.66-24.38）	2.41（1.7-3.28）	-0.41 (-1.22 to 0.4)
Italy	22.36（20.22-24.53）	0.48（0.43-0.53）	146.41（124.35-166.03）	1.65（1.41-1.87）	4.17 (2.53 to 5.83)
Jamaica	3.42（2.28-4.82）	3.27（2.17-4.61）	6.94（4.51-10.17）	3.83（2.48-5.62）	0.62 (-0.57 to 1.82)
Japan	40.16（37.56-42.38）	0.49（0.46-0.52）	106.94（94.1-115.33）	0.45（0.4-0.48）	-0.28 (-0.78 to 0.22)
Jordan	1.33（0.67-2.4）	2.15（1.06-3.92）	6.35（3.22-11.34）	1.76（0.89-3.15）	-0.6 (-0.89 to -0.3)
Kazakhstan	1.74（1.12-2.48）	0.39（0.24-0.56）	3.1（2.16-4.25）	0.44（0.3-0.6）	0.36 (-0.26 to 0.98)
Kenya	40.21（26.32-77.55）	10.07（6.6-19.29）	148.3（79.72-281.33）	15.92（8.59-29.85）	1.49 (1.39 to 1.58)
Kiribati	0.03（0.01-0.04）	1.62（0.83-2.79）	0.05（0.03-0.09）	1.78（0.86-3.17）	0.33 (0.22 to 0.43)

Kuwait	0.41（0.29-0.56）	1.51（1.06-2.1）	1.32（0.89-1.9）	0.88（0.59-1.27）	-0.84 (-2.44 to 0.77)
Kyrgyzstan	0.64（0.46-0.88）	0.59（0.42-0.81）	3.63（2.54-5.02）	1.9（1.32-2.62）	2.97 (-0.61 to 6.69)
Lao People's Democratic Republic	1.3（0.58-2.58）	1.3（0.57-2.57）	3.9（1.66-7.31）	1.84（0.79-3.42）	1.14 (1.01 to 1.27)
Latvia	0.43（0.31-0.57）	0.31（0.22-0.41）	1.48（1.01-2.06）	0.81（0.55-1.12）	3.31 (2.14 to 4.48)
Lebanon	5.17（2.57-9.24）	4.85（2.4-8.66）	16.92（8.21-30.64）	4.6（2.25-8.32）	-0.15 (-0.44 to 0.13)
Lesotho	1.08（0.54-1.97）	3.65（1.8-6.74）	2.14（1.03-3.77）	5.39（2.56-9.56）	1.41 (1.14 to 1.68)
Liberia	1.94（0.93-3.67）	3.01（1.42-5.71）	2.49（1.03-5.75）	2.66（1.1-6.08）	-0.43 (-0.69 to -0.17)
Libya	1.19（0.57-2.21）	1.11（0.53-2.09）	3.53（1.41-6.88）	1.35（0.54-2.66）	0.67 (0.19 to 1.16)
Lithuania	1.42（1.04-1.89）	0.71（0.52-0.95）	2.57（1.82-3.51）	0.95（0.68-1.3）	0.87 (-0.13 to 1.88)
Luxembourg	0.38（0.32-0.45）	1.47（1.23-1.73）	1.04（0.85-1.25）	1.72（1.39-2.06）	0.48 (-0.87 to 1.85)
Madagascar	24.11（11.96-45.55）	9.19（4.58-17.38）	40.37（18.71-77.46）	9.19（4.28-17.62）	-0.01 (-0.19 to 0.16)
Malawi	22.13（10.17-41.96）	12.94（5.9-24.89）	47.28（19.98-92.23）	16.13（6.8-31.68）	0.74 (0.6 to 0.88)
Malaysia	4.06（1.03-7.7）	0.85（0.21-1.62）	18.03（4.01-33.6）	1.15（0.25-2.16）	0.92 (0.6 to 1.24)
Maldives	0.05（0.02-0.09）	0.92（0.44-1.71）	0.12（0.06-0.21）	0.71（0.37-1.23）	-0.89 (-1.09 to -0.68)
Mali	4.91（2.2-10.15）	2.59（1.14-5.31）	8.99（4.27-19.41）	2.2（1.03-4.68）	-0.48 (-0.63 to -0.33)
Malta	0.19（0.14-0.25）	0.85（0.62-1.12）	0.56（0.39-0.76）	0.91（0.64-1.24）	-0.1 (-1.46 to 1.29)
Marshall Islands	0.01（0-0.01）	0.77（0.32-1.73）	0.02（0.01-0.04）	1.21（0.52-2.5）	1.5 (1.35 to 1.65)
Mauritania	1.35（0.66-2.5）	2.92（1.4-5.42）	2.58（0.89-6.02）	2.36（0.82-5.4）	-0.67 (-0.77 to -0.57)
Mauritius	0.39（0.33-0.47）	1.18（0.98-1.41）	2.6（2.15-3.11）	2.6（2.14-3.1）	2.49 (-6.9 to 12.84)
Mexico	11（10.32-11.66）	0.5（0.47-0.53）	56.9（47.32-67.25）	0.83（0.69-0.98）	1.72 (0.11 to 3.36)
Micronesia (Federated States of)	0.02（0.01-0.05）	0.88（0.41-1.73）	0.05（0.02-0.09）	1.46（0.7-2.63）	1.65 (1.53 to 1.77)
Monaco	0.14（0.07-0.24）	3.46（1.73-6.05）	0.2（0.1-0.35）	3.18（1.66-5.61）	-0.28 (-0.36 to -0.2)
Mongolia	0.1（0.05-0.19）	0.2（0.1-0.4）	0.3（0.16-0.51）	0.34（0.19-0.58）	1.69 (1.06 to 2.33)
Montenegro	0.45（0.22-0.83）	1.51（0.75-2.79）	1.22（0.58-2.17）	2.52（1.2-4.46）	1.93 (1.21 to

					2.65)
Morocco	7.31（3.53-13.5）	0.88（0.42-1.66）	25.11（12.63-45.02）	1.29（0.64-2.35）	1.22 (1.07 to 1.37)
Mozambique	35.09（17.18-66.19）	13.41（6.52-25.45）	82.52（41.12-146.98）	19.6（9.64-35.63）	1.25 (1.13 to 1.37)
Myanmar	12.52（5.93-22.99）	1.14（0.54-2.09）	38.87（16.67-70.62）	1.81（0.78-3.28）	1.51 (1.43 to 1.59)
Namibia	1.95（1.07-3.41）	7.09（3.77-12.58）	4.82（2.59-8.38）	9.2（4.94-16.08）	0.88 (0.78 to 0.98)
Nauru	0（0-0.01）	1.18（0.56-2.18）	0（0-0.01）	1.93（0.86-3.59）	1.61 (1.54 to 1.69)
Nepal	5.27（2.55-10.47）	1.19（0.57-2.36）	29.62（14.29-55.13）	2.59（1.25-4.82）	2.58 (2.46 to 2.7)
Netherlands	11.78（9.06-15.02）	1.18（0.91-1.51）	29.32（21.38-38.57）	1.39（1.01-1.83）	0.65 (0.16 to 1.15)
New Zealand	4.38（3.25-5.77）	2.14（1.59-2.82）	6.84（4.91-9.17）	1.33（0.95-1.78）	-0.93 (-2.12 to 0.27)
Nicaragua	0.56（0.34-0.82）	0.78（0.47-1.14）	1.71（1.11-2.58）	0.71（0.46-1.07）	-0.39 (-0.62 to -0.15)
Niger	2.8（1.25-5.88）	2.07（0.9-4.32）	5.7（2.27-13.29）	1.59（0.64-3.65）	-0.84 (-1.01 to -0.67)
Nigeria	57.18（31.52-89.66）	2.71（1.48-4.14）	89.27（48.3-167.04）	2.38（1.27-4.29）	-0.42 (-0.46 to -0.38)
Niue	0（0-0）	0.92（0.46-1.63）	0（0-0）	1.71（0.86-3）	2.04 (1.99 to 2.09)
North Macedonia	2.5（1.74-3.86）	2.58（1.8-4.02）	7.02（4.04-10.36）	4.2（2.44-6.17）	1.62 (1.14 to 2.1)
Northern Mariana Islands	0（0-0.01）	0.34（0.13-1.14）	0.01（0.01-0.03）	0.49（0.23-1.29）	1.09 (0.7 to 1.47)
Norway	4.71（4.17-5.28）	1.24（1.09-1.39）	6.49（5.46-7.47）	1.04（0.87-1.2）	-0.21 (-2.02 to 1.62)
Oman	0.74（0.29-1.47）	2.4（0.92-4.78）	2.25（1.02-4.18）	3.19（1.39-5.92）	0.96 (-0.2 to 2.14)
Pakistan	109（66.4-171.75）	3.21（1.94-5.1）	263.61（153.85-417.27）	4.45（2.61-7.06）	1.06 (0.98 to 1.15)
Palau	0（0-0.01）	0.84（0.41-1.54）	0.01（0.01-0.02）	0.82（0.41-1.49）	-0.14 (-0.3 to 0.01)
Palestine	0.75（0.37-1.37）	1.83（0.9-3.35）	1.8（0.98-3.09）	1.71（0.93-2.96）	-0.29 (-0.75 to 0.18)
Panama	0.91（0.74-1.11）	1.07（0.87-1.3）	2.14（1.54-2.79）	0.82（0.59-1.06）	-0.93 (-1.55 to -0.3)
Papua New Guinea	0.33（0.11-0.8）	0.37（0.12-0.88）	1.4（0.46-3.42）	0.54（0.18-1.31）	1.27 (1.08 to 1.46)
Paraguay	0.36（0.19-0.61）	0.3（0.16-0.51）	1.78（0.9-3.11）	0.55（0.28-0.97）	2.08 (1.61 to 2.55)
Peru	5.19（2.7-9.08）	0.8（0.41-1.39）	19.12（9.81-33.19）	0.99（0.51-1.72）	0.84 (-0.44 to 2.14)
Philippines	20.71（13.03-	1.56（0.98-	80.62（48.96-	2.12（1.28-	1.1

	26.84）	2.02）	106.24）	2.77）	(0.85 to 1.36)
Poland	23.67（21.94-25.37）	1.22（1.12-1.31）	83.88（73.02-94.61）	2.24（1.94-2.52）	1.93 (0.23 to 3.66)
Portugal	12.4（9.37-16.32）	1.82（1.37-2.4）	30.55（21.78-41.82）	2.1（1.51-2.86）	0.51 (-0.65 to 1.69)
Puerto Rico	1.88（1.34-2.58）	0.93（0.66-1.28）	8.63（5.91-11.98）	1.94（1.33-2.69）	2.57 (-0.71 to 5.96)
Qatar	0.05（0.02-0.09）	1.15（0.54-2.15）	0.26（0.12-0.53）	0.88（0.41-1.76）	-1.19 (-2.59 to 0.22)
Republic of Korea	8.78（4.65-14.57）	0.87（0.46-1.46）	16.5（9.22-28.26）	0.35（0.19-0.6）	-3 (-3.15 to -2.84)
Republic of Moldova	5.32（4.24-6.51）	2.81（2.25-3.44）	5.64（4.6-6.83）	1.85（1.51-2.24）	-1.41 (-3.23 to 0.44)
Romania	28.37（19.4-39.26）	2.04（1.4-2.82）	45.34（32.22-61.64）	2.2（1.56-2.99）	0.17 (-0.66 to 1.02)
Russian Federation	197.55（184.03-211.45）	3.18（2.94-3.42）	153.21（131.39-173.33）	1.37（1.17-1.55）	-2.64 (-4.21 to -1.05)
Rwanda	20.42（9.41-37.89）	16.46（7.58-30.69）	32.9（13.45-66.55）	14.36（5.89-28.83）	-0.45 (-0.55 to -0.34)
Saint Kitts and Nevis	0.25（0.2-0.31）	13.65（10.81-16.64）	0.45（0.32-0.63）	14.97（10.95-20.57）	0.37 (-1.24 to 2.01)
Saint Lucia	0.14（0.11-0.17）	3.57（2.82-4.4）	0.8（0.61-1.03）	6.29（4.81-8.09）	2.1 (-1.15 to 5.44)
Saint Vincent and the Grenadines	0.02（0.02-0.03）	0.68（0.56-0.82）	0.28（0.23-0.35）	3.49（2.83-4.22）	5.58 (2.77 to 8.47)
Samoa	0.04（0.02-0.08）	1.02（0.51-1.79）	0.1（0.05-0.18）	1.29（0.64-2.31）	0.77 (0.63 to 0.9)
San Marino	0.01（0-0.01）	0.44（0.24-0.75）	0.01（0.01-0.02）	0.21（0.1-0.4）	-2.73 (-3.13 to -2.33)
Sao Tome and Principe	0.08（0.04-0.16）	2.92（1.38-5.64）	0.13（0.06-0.27）	2.83（1.25-5.63）	-0.17 (-0.48 to 0.14)
Saudi Arabia	4.02（1.96-7.44）	1.35（0.65-2.5）	14.52（6.62-27.34）	1.8（0.84-3.37）	0.93 (0.84 to 1.03)
Senegal	4.72（2.25-9.28）	2.79（1.32-5.47）	9.29（4.23-19.31）	2.61（1.19-5.33）	-0.25 (-0.4 to -0.09)
Serbia	17.15（9.3-28.68）	3.53（1.9-5.91）	32.13（17.34-54.68）	3.4（1.82-5.81）	0 (-0.15 to 0.16)
Seychelles	0.11（0.06-0.2）	3.9（1.95-7.09）	0.2（0.1-0.35）	3.67（1.89-6.48）	-0.29 (-1.52 to 0.96)
Sierra Leone	2.88（1.34-5.53）	2.53（1.17-4.87）	3.6（1.74-7.27）	2.02（0.98-4.04）	-0.72 (-0.78 to -0.65)
Singapore	0.53（0.4-0.69）	0.51（0.38-0.67）	1.54（1.11-2.09）	0.32（0.23-0.44）	-1.42 (-1.97 to -0.87)
Slovakia	5.48（3.13-8.96）	1.81（1.03-2.97）	10.14（5.24-17.82）	2.08（1.08-3.66）	0.55 (0.19 to 0.91)
Slovenia	0.91（0.68-1.19）	0.81（0.6-1.06）	5.01（3.57-6.81）	2（1.42-2.72）	3.02 (2.2 to 3.84)

Solomon Islands	0.04（0.01-0.09）	0.53（0.19-1.19）	0.15（0.06-0.34）	0.9（0.36-1.98）	1.74 (1.54 to 1.94)
Somalia	12.7（5.78-24.7）	14.29（6.51-27.99）	29.78（13.87-60.83）	14（6.39-28.91）	-0.05 (-0.13 to 0.04)
South Africa	19.06（13.71-28.27）	2.29（1.64-3.35）	63.3（43.78-81.73）	3.51（2.41-4.51）	1.38 (1.04 to 1.72)
South Sudan	20.97（9.89-40.47）	12.99（6.13-25.19）	28.17（11.91-54.64）	15.64（6.7-30.45）	0.6 (0.53 to 0.68)
Spain	32.51（25.28-40.93）	1.13（0.88-1.43）	86.75（62.27-116.7）	1.48（1.07-1.98）	1.02 (0.3 to 1.73)
Sri Lanka	8.06（4.23-13.71）	1.47（0.77-2.54）	21.92（10.15-39.42）	1.65（0.78-2.96）	0.48 (-0.53 to 1.5)
Sudan	4.29（1.73-9.33）	0.77（0.31-1.69）	11.3（5.61-20.54）	1.02（0.5-1.88）	0.89 (0.81 to 0.96)
Suriname	0.27（0.14-0.46）	1.96（1.05-3.36）	0.88（0.42-1.6）	2.68（1.28-4.9）	1.13 (0.3 to 1.98)
Sweden	7.77（5.83-10）	0.9（0.68-1.16）	8.88（6.25-12.16）	0.63（0.44-0.86）	-0.88 (-1.91 to 0.16)
Switzerland	7.51（5.42-9.96）	1.42（1.03-1.88）	7.36（5.01-10.27）	0.65（0.45-0.91）	-2.38 (-3.91 to -0.81)
Syrian Arab Republic	0.31（0.15-0.59）	0.1（0.05-0.2）	1（0.49-1.82）	0.14（0.07-0.25）	0.85 (0.43 to 1.28)
Taiwan (Province of China)	4.17（3.22-5.31）	0.41（0.32-0.53）	18.04（13.1-24.26）	0.68（0.49-0.91）	1.17 (0.08 to 2.27)
Tajikistan	0.28（0.12-0.61）	0.23（0.1-0.51）	0.85（0.35-1.82）	0.33（0.14-0.69）	1.12 (0.49 to 1.75)
Thailand	10.06（5.36-17.34）	0.65（0.35-1.13）	39.08（19.92-65.2）	0.63（0.32-1.06）	-0.06 (-0.37 to 0.26)
Timor-Leste	0.08（0.04-0.16）	0.72（0.33-1.38）	0.58（0.25-1.11）	1.28（0.54-2.42）	1.87 (1.79 to 1.94)
Togo	1.44（0.67-2.89）	2.6（1.19-5.21）	3.57（1.78-6.91）	2.71（1.34-5.2）	0.14 (0.02 to 0.26)
Tokelau	0（0-0）	0.67（0.29-1.35）	0（0-0）	1.14（0.55-2.12）	1.74 (1.61 to 1.86)
Tonga	0.02（0.01-0.04）	0.66（0.31-1.29）	0.05（0.03-0.1）	1.27（0.6-2.32）	2.14 (1.74 to 2.54)
Trinidad and Tobago	2.14（1.78-2.57）	5.09（4.22-6.12）	4.03（2.86-5.49）	3.58（2.56-4.85）	-1 (-2.85 to 0.88)
Tunisia	7.92（3.95-14.27）	2.97（1.49-5.35）	25.85（11.11-48.58）	3.64（1.58-6.83）	0.65 (0.37 to 0.94)
Turkey	33.53（16.94-62.92）	2.03（1.01-3.81）	91（48.31-155.14）	1.83（0.97-3.14）	-0.36 (-0.75 to 0.02)
Turkmenistan	0.48（0.32-0.65）	0.64（0.43-0.89）	1.81（1.15-2.78）	1.09（0.7-1.66）	1.74 (1.43 to 2.06)
Tuvalu	0（0-0.01）	0.7（0.28-1.55）	0.01（0-0.01）	1.2（0.55-2.23）	1.77 (1.71 to 1.84)
Uganda	52.16（27.49-89.58）	16.87（8.83-29.1）	116（58.15-205.33）	21.17（10.65-37.55）	0.75 (0.62 to

					0.88)
Ukraine	54.46（38.25-74.21）	1.72（1.21-2.36）	55.46（32.77-85.24）	1.42（0.84-2.17）	-0.66 (-1.3 to -0.01)
United Arab Emirates	0.12（0.05-0.26）	0.68（0.29-1.46）	0.95（0.49-1.7）	0.6（0.31-1.09）	-0.71 (-1.73 to 0.32)
United Kingdom	84.82（80.53-88.6）	1.83（1.73-1.92）	77.83（70.82-83）	0.95（0.87-1.02）	-2.03 (-3.21 to -0.83)
United Republic of Tanzania	69.96（34.17-129.37）	13.29（6.46-24.75）	145.6（64.89-299.13）	12.71（5.7-26.01）	-0.13 (-0.23 to -0.04)
United States of America	0.09（0.05-0.18）	1.72（1.61-1.81）	0.34（0.17-0.62）	1.42（1.29-1.51）	1.19 (0.43 to 1.95)
United States Virgin Islands	293.77（276.82-307.56）	2.53（1.27-4.93）	498.03（454.43-529.27）	3.54（1.79-6.38）	-0.56 (-1.32 to 0.2)
Uruguay	9.13（6.51-12.36）	4.34（3.09-5.88）	9.56（6.95-13.02）	3.17（2.31-4.32）	-1.08 (-1.82 to -0.33)
Uzbekistan	1.11（0.53-1.7）	0.24（0.11-0.36）	3.11（2.21-4.34）	0.28（0.2-0.39）	0.67 (-1.29 to 2.66)
Vanuatu	0.02（0.01-0.04）	0.55（0.21-1.23）	0.09（0.04-0.18）	0.97（0.41-2.03）	1.85 (1.75 to 1.96)
Venezuela (Bolivarian Republic of)	3.47（2.85-4.16）	0.7（0.57-0.84）	16.98（11.89-23.03）	1.04（0.73-1.4）	1.37 (-0.18 to 2.95)
Viet Nam	32.87（17.7-55.22）	1.89（1.01-3.2）	122.97（62.87-213.03）	3.03（1.52-5.26）	1.57 (1.45 to 1.7)
Yemen	1.96（0.73-4.46）	0.8（0.29-1.83）	5.57（2.25-12.05）	0.78（0.31-1.7）	-0.08 (-0.21 to 0.04)
Zambia	20.99（10.36-38.37）	14.35（7.02-26.39）	51.5（19.54-128.58）	18.19（7.03-43.49）	0.86 (0.65 to 1.08)
Zimbabwe	3（1.56-5.32）	1.76（0.91-3.11）	6.48（3.43-11.2）	2.74（1.4-4.81）	1.54 (1.26 to 1.82)

Abbreviations: SDI: Sociodemographic Index, ASIR: age-standardised incidence rate, ASMR: age-standardised mortality rate, ASDR: age-standardised disability-adjusted life years rate; AAPC=average annual percentage change; CI = confidence interval; UI = uncertainty interval.

Table S3. The DALYs and ASDR of older males with breast cancer in 1990 and 2021, and its AAPC from 1990 to 2021

Characteristics	1990		2021		1990-2021
	DALYs (95 % UI)	ASDR (95 % UI)	DALYs (95 % UI)	ASDR (95 % UI)	AAPC (95 % CI)
Global	70599.93（60676.63-85390.63）	32.85（28.24-39.72）	199594.32（137777.3-243992.47）	39.68（27.61-48.39）	0.68 (0.43 to 0.92)
High SDI	16825.3（15734.11-17872.11）	28.56（26.59-30.39）	31550.11（28535.76-34634.65）	25.14（22.75-27.59）	-0.37 (-0.93 to 0.2)
High-middle SDI	16249.7（14352.9-18528.44）	31.23（27.53-35.6）	40808.91（27238.7-51278.3）	35.6（24.11-44.48）	0.48 (0.05 to 0.91)
Low SDI	13513.98（9904.36-19924.31）	104.29（76.44-154.97）	26156.4（19158.61-41679.2）	99.21（72.64-157.23）	-0.15 (-0.24 to -0.06)
Low-middle SDI	10258.86（7919.48-14199.85）	30.04（23.06-41.99）	36480.88（25525.5-47037.55）	46.06（32.21-59.47）	1.43 (1.22 to 1.64)
Middle SDI	13675.46（10033.95-17420.43）	24.03（17.68-30.52）	64403.6（31997.96-84477.27）	41.03（20.71-53.57）	1.75 (1.56 to 1.93)
Region					
Andean Latin America	145.82（89.16-235.97）	6.55（4.92-8.14）	558.69（340.6-867.99）	16.41（10.01-25.51）	1.04 (0.07 to 2.02)
Australasia	408.28（325.03-500.71）	30.66（24.35-37.68）	797.21（604.82-1023.21）	23.91（18.18-30.66）	-0.43 (-1.9 to 1.06)

Caribbean	411.4（330.3-534.25）	10.31（9.17-11.66）	1359（1068.63-1714.46）	43.74（34.39-55.21）	1.56 (0.17 to 2.96)
Central Asia	133.62（102.11-165.64）	32.42（27.92-37.57）	527.98（452.61-612.38）	13.58（11.66-15.72）	1.97 (0.02 to 3.97)
Central Europe	2537.12（2187.93-2933.27）	20.99（14.78-30.09）	5902.16（5083.86-6733.22）	47.03（40.46-53.69）	1.3 (0.61 to 1.99)
Central Latin America	502.66（462.37-544.47）	11.64（6.12-20.76）	2370.74（2041.18-2739.35）	16.8（14.46-19.4）	1.46 (0.47 to 2.46)
Central Sub-Saharan Africa	906.75（502.61-1604.5）	38.81（36.11-41.63）	1951.56（1100.38-3573.3）	77.34（43.33-143.66）	0.01 (-0.13 to 0.15)
East Asia	10883.73（7641.04-15454.27）	76.98（42.14-138.35）	54341.02（21878.44-78791.79）	39.58（16.37-57.14）	2.06 (1.68 to 2.45)
Eastern Europe	6265.38（5673.39-6860.02）	51.75（29.62-78.37）	5222.23（4402.88-6124.58）	29.08（24.54-34.06）	-1.78 (-2.65 to -0.9)
Eastern Sub-Saharan Africa	11234.57（8215.78-17659.7）	52.43（47.41-57.5）	22124.34（14285.88-39275.65）	269.29（174.93-476.59）	-0.01 (-0.05 to 0.03)
High-income Asia Pacific	1055.14（939.96-1196.56）	24.52（21.85-27.34）	2341.47（2044.94-2671.9）	8.41（7.35-9.62）	-0.78 (-1.69 to 0.14)
High-income North America	7569.9（7065.03-8110.24）	12.75（7.79-20.64）	12425.55（11392.47-13490.21）	30.8（28.18-33.46）	-0.63 (-1.42 to 0.16)
North Africa and Middle East	2796.39（1912.19-4158.07）	10.92（10.02-11.83）	8614.4（6068.79-12273.15）	33.93（23.77-48.47）	0.5 (0.4 to 0.61)
Oceania	19.6（10.17-35.3）	270.59（197.39-428.97）	66.95（32.71-126.28）	15.68（7.75-29.38）	1 (0.68 to 1.31)
South Asia	8937.49（6689.91-12490.74）	27.16（21.78-35.17）	36765.41（21921.79-47635.58）	44.63（26.67-57.81）	1.61 (1.21 to 2.01)
Southeast Asia	2808.07（1834.6-3639.37）	21.67（14.1-28.11）	11745.19（6143.79-15349.54）	33.49（17.54-43.73）	1.42 (1.33 to 1.52)
Southern Latin America	1187.68（899.89-1544.39）	43.39（31.9-62.11）	2102.62（1652.57-2620.56）	43.09（33.81-53.77）	-0.11 (-1.02 to 0.82)
Southern Sub-Saharan Africa	539.54（398.54-776.73）	46.52（35.16-60.62）	1708.83（1187.51-2165.95）	66.7（45.97-84.21）	1.39 (1.13 to 1.65)
Tropical Latin America	1246.64（1115.49-1387.74）	27.8（20.64-39.14）	6744（5994.59-7506.5）	46.28（41.02-51.57）	2.09 (0.79 to 3.41)
Western Europe	8438.29（7705.21-9165.8）	29.19（19.91-43.27）	17569.53（15125.1-20052.72）	31.95（27.57-36.45）	0.58 (-0.4 to 1.57)
Western Sub-Saharan Africa	2571.87（1490.27-3930.58）	27.72（25.24-30.16）	4355.41（2803.15-7707.05）	45.15（28.47-78.4）	-0.43 (-0.48 to -0.39)
204 countries and territories					
Afghanistan	115.93（44.62-261.14）	24.22（9.32-54.7）	71.95（31.3-149.81）	20.52（8.83-42.8）	-0.54 (-0.61 to -0.48)
Albania	41.51（24.8-66.62）	41.21（24.44-66.63）	123.48（60-233.86）	45.47（22.2-86.05）	0.29 (-0.16 to 0.73)
Algeria	342.18（185.96-582.06）	53.68（28.86-91.28）	1338.68（606.37-2452.75）	68.31（30.6-125.64）	0.8 (0.68 to 0.92)
American Samoa	0.22（0.11-0.38）	17.74（9.18-	0.57（0.32-0.97）	20.05（11.05-	0.51 (-0.55 to

		31.31）		34.18）	1.58)
Andorra	0.94（0.47-1.75）	26.02（13.04-48.09）	1.7（0.81-3.1）	17.43（8.32-31.94）	-1.32 (-2.13 to -0.51)
Angola	154.36（74.21-303.33）	74.99（35.56-147.01）	437.42（214.81-796.7）	81.82（39.92-150.22）	0.29 (0.06 to 0.52)
Antigua and Barbuda	0.81（0.67-0.98）	27.27（22.45-33.06）	2.7（2.12-3.49）	44.74（35.16-57.63）	1.46 (-1.7 to 4.72)
Argentina	974.07（701.52-1330.62）	54.37（39.04-74.39）	1584.81（1171.64-2086.28）	50.87（37.55-67.06）	0.03 (-0.93 to 1)
Armenia	18.07（12.56-24.84）	14.05（9.65-19.34）	55.23（40.21-74.06）	22.86（16.61-30.72）	1.15 (-1.35 to 3.73)
Australia	315.42（241.85-403.66）	28.46（21.77-36.47）	669.31（486.69-888.7）	23.8（17.33-31.58）	-0.38 (-1.84 to 1.09)
Austria	161.58（121.55-210.85）	28.29（21.28-36.87）	380.51（270.84-511.86）	36.54（26.06-49.06）	1.01 (0.13 to 1.91)
Azerbaijan	21.58（11.33-37.44）	10.52（5.51-18.22）	59.98（31.02-107.22）	11.91（6.14-21.33）	0.41 (0.17 to 0.64)
Bahamas	7.02（5.65-8.58）	93.77（75.64-114.61）	18.68（13.97-24.12）	86.82（65.3-111.82）	-0.22 (-2.38 to 1.98)
Bahrain	2.48（1.3-4.26）	29.1（15.18-50.43）	12.62（5.91-25.08）	29.58（14.28-56.51）	0.03 (-0.49 to 0.55)
Bangladesh	812.25（396.46-1587.81）	27.2（13.24-53.14）	2837.88（1361.57-5563.62）	33.63（16.23-65.55）	0.74 (0.09 to 1.4)
Barbados	10.53（8.58-12.88）	66.93（54.47-81.83）	18.3（13.36-24.38）	59.3（43.39-78.97）	-0.24 (-1.42 to 0.95)
Belarus	87.5（63.5-116.65）	15.8（11.38-21.19）	8.2（5.63-11.53）	1.03（0.7-1.46）	-8.98 (-10.91 to -7)
Belgium	263.88（193.91-350.13）	32.28（23.71-42.85）	525.22（369.05-713.8）	38.04（26.76-51.66）	0.45 (-0.48 to 1.39)
Belize	3.16（1.9-4.27）	56.19（33.8-76.05）	13.27（10.47-16.55）	76.05（60-94.75）	1.34 (-1.4 to 4.16)
Benin	62.37（32.06-112.88）	55.74（28.53-100.84）	116.05（53.72-238.88）	46.77（21.67-95.03）	-0.57 (-0.77 to -0.38)
Bermuda	0.86（0.59-1.29）	27.43（18.85-40.61）	3.43（2.36-4.89）	43.23（29.74-61.54）	1.28 (-0.86 to 3.47)
Bhutan	3.42（1.52-6.98）	28.28（12.51-58.82）	14.95（6.33-29.49）	42.43（18-83.59）	1.34 (1.23 to 1.45)
Bolivia (Plurinational State of)	25.37（12.05-51.01）	14.86（7.04-29.81）	103.41（50.39-188.48）	20.2（9.88-36.73）	0.98 (0.78 to 1.19)
Bosnia and Herzegovina	53.44（26.13-100.76）	27.55（13.41-52.16）	124.61（65.13-211.21）	34.08（17.77-57.71）	0.73 (0.29 to 1.16)
Botswana	18.46（9.08-33.93）	69.51（33.77-128.33）	46.84（23.83-84.53）	75.1（38.3-134.73）	0.33 (-0.13 to 0.79)
Brazil	1238.85（1109.26-1379.6）	25.01（22.29-27.9）	6704.23（5950.04-7459.99）	47.11（41.69-52.49）	2.08 (0.78 to 3.41)
Brunei Darussalam	1.47（0.75-2.71）	27.29（13.91-50.21）	2.54（1.36-4.79）	15.27（8.14-28.78）	-1.96 (-3.26 to -0.65)

Bulgaria	163.7（123.27-209.61）	22.88（17.23-29.42）	385.29（266.26-539.15）	48.55（33.61-67.86）	2.38 (1.21 to 3.56)
Burkina Faso	127.64（57.21-247.78）	52.45（23.28-102.56）	208.29（100.41-401.71）	44.68（21.43-85.99）	-0.5 (-0.69 to -0.31)
Burundi	353.11（176.26-650.25）	291.83（145.21-538.32）	614.72（291.28-1204.96）	233.01（110.41-456.46）	-0.75 (-0.88 to -0.63)
Cabo Verde	3.95（1.64-10.25）	30.37（12.64-78.86）	12.24（5.46-24.77）	56.01（25.15-111.96）	1.93 (0.59 to 3.28)
Cambodia	52.87（25.46-97.55）	25.05（12-46.26）	240.83（100.16-445.53）	43.32（18.17-80.39）	1.79 (1.71 to 1.87)
Cameroon	184.17（91.61-334.84）	75.12（37.15-136.62）	404.4（179.38-787.15）	63.77（28.01-124.21）	-0.53 (-0.64 to -0.42)
Canada	413.95（316.18-533.39）	22.73（17.34-29.32）	879.41（643.31-1175.55）	19.58（14.32-26.18）	-0.06 (-1.31 to 1.2)
Central African Republic	55.22（25.26-113.76）	91.64（41.46-189.79）	84.54（39.27-175.09）	85.27（38.71-174.35）	-0.25 (-0.32 to -0.19)
Chad	57.57（24.08-122.01）	36.79（15.31-78.13）	123.38（55.73-248.93）	36.13（16.27-73.07）	-0.05 (-0.22 to 0.11)
Chile	22.26（16.54-29.6）	4.11（3.04-5.47）	329.56（240.97-441.04）	22.55（16.46-30.22）	5.3 (2.14 to 8.55)
China	10683.61（7466.54-15227.45）	21.37（14.98-30.78）	53492.46（21253.69-77929.74）	40.29（16.46-58.44）	2.06 (1.67 to 2.46)
Colombia	90.39（68.58-116.84）	9.13（6.91-11.83）	448.87（312.93-621.34）	14.51（10.11-20.11）	1.92 (0.84 to 3.02)
Comoros	23.49（9.21-45.21）	221.03（87.2-427.84）	53.22（16.99-117.9）	225.09（72.1-497.19）	0.03 (-0.08 to 0.13)
Congo	59.8（30.73-104.76）	112.78（57.59-198.54）	125.75（64.25-223.49）	97.18（49.51-175.16）	-0.47 (-0.57 to -0.37)
Cook Islands	0.14（0.07-0.24）	17.87（9.45-30.48）	0.42（0.2-0.75）	25.53（12.1-45.63）	1.17 (1.07 to 1.27)
Costa Rica	4.13（3-5.51）	4.16（3.02-5.55）	58.62（42.12-79.89）	18.35（13.18-25.04）	5.26 (4.23 to 6.31)
Coted'Ivoire	207.25（109.92-368.38）	92.05（48.58-163.69）	562.84（268.44-1098.01）	93.3（44.37-179.88）	0.04 (-0.05 to 0.13)
Croatia	128.13（97.26-165.48）	47.04（35.66-60.92）	351.35（255.61-474.73）	69.16（50.3-93.47）	1.3 (0.44 to 2.17)
Cuba	89.38（63.55-122.16）	14.46（10.3-19.73）	364.86（249.98-511.45）	32.37（22.17-45.36）	2.96 (1.59 to 4.35)
Cyprus	24.9（13.12-43.06）	67.25（34.73-118.08）	49.03（26.63-83.57）	39.64（21.51-67.52）	-1.79 (-2 to -1.57)
Czechia	121.92（92.91-156.34）	17.11（13.06-21.94）	459.73（331.12-622.32）	37.3（26.83-50.63）	2.61 (1.75 to 3.48)
Democratic People's Republic of Korea	100.47（48.95-185.7）	14.76（7.22-27.3）	417.04（166.79-805.32）	25.08（10.09-48.41）	1.72 (1.59 to 1.85)
Democratic Republic of the Congo	593.65（273.57-1178.14）	72.44（32.89-145.84）	1209.51（529.09-2537.76）	71.55（31.03-152.09）	-0.05 (-0.22 to 0.12)

Denmark	143.94（106.11-189.65）	32.05（23.61-42.22）	165.06（116.37-226.7）	22.56（15.92-30.98）	-1.21 (-1.77 to -0.65)
Djibouti	13.66（6.39-27.15）	213.51（100.67-421.8）	87.26（36.21-178.7）	264.17（111.07-539.45）	0.68 (0.62 to 0.74)
Dominica	1.39（0.73-2.47）	46.07（24.18-82）	2.73（1.47-4.66）	55.51（29.96-94.92）	0.63 (0.51 to 0.75)
Dominican Republic	39.63（20.33-76.94）	18.98（9.73-37.05）	264.48（133.97-471.65）	46.04（23.29-82.18）	2.92 (2.11 to 3.74)
Ecuador	12.94（9.49-17.39）	4.39（3.22-5.91）	65.6（42.27-97.66）	7.04（4.56-10.44）	0.89 (-1.32 to 3.15)
Egypt	327.06（148.33-665.32）	21.28（9.59-43.52）	1126.36（622.79-2146.25）	28.92（15.81-55.56）	1.03 (0.69 to 1.37)
El Salvador	27.31（17.03-39.49）	16.55（10.32-23.96）	57.01（36.89-84.65）	17.83（11.53-26.45）	0.44 (-0.36 to 1.23)
Equatorial Guinea	8.9（4.16-18.09）	91.1（42.34-184.31）	22.74（10.01-42.46）	106.95（47.35-200.07）	0.54 (0.37 to 0.71)
Eritrea	102.47（48.91-205.91）	241.24（114.18-483.53）	250.58（118.11-485.23）	248.2（116.97-480.62）	0.08 (-0.02 to 0.17)
Estonia	8.01（5.94-10.53）	9.32（6.9-12.25）	22.56（15.75-30.98）	17.22（12.03-23.63）	2.02 (-0.02 to 4.11)
Eswatini	13.9（7.47-24.37）	114.07（60.52-200.23）	31.79（14.98-57.29）	140.83（65.74-256.89）	0.7 (0.54 to 0.86)
Ethiopia	3882.41（2441.05-6317.15）	326.88（204.61-536.23）	5389.61（3222.15-10545.55）	229.53（137.57-445.75）	-1.15 (-1.26 to -1.04)
Fiji	5.42（2.55-9.95）	28.91（13.68-53.17）	15.62（6.99-28.76）	36.64（16.56-67.21）	0.75 (0.14 to 1.36)
Finland	70.69（52.17-92.57）	20.52（15.13-26.94）	70.05（49.75-97.75）	9.19（6.53-12.8）	-2.57 (-4.11 to -1)
France	1973.12（1576.11-2377.24）	45.24（36.15-54.55）	4499.71（3182.25-6212.59）	55.48（39.31-76.52）	0.88 (-0.53 to 2.3)
Gabon	34.82（17.34-63.65）	112.6（55.99-206.48）	71.6（35.71-126.66）	129.15（64.19-230.02）	0.47 (0.29 to 0.64)
Gambia	10.77（5.1-20.34）	54.28（25.56-102.5）	29.01（14.32-55.5）	58.29（28.45-111.71）	0.23 (-0.3 to 0.76)
Georgia	0.36（0.23-0.57）	0.14（0.09-0.23）	119.52（98.33-143.77）	38.54（31.71-46.32）	19.11 (12.29 to 26.33)
Germany	1601.41（1208.61-2081.8）	27.39（20.64-35.59）	3410.53（2541.8-4403.31）	29.78（22.26-38.41）	0.37 (-0.77 to 1.53)
Ghana	117.03（51.21-214.29）	33.11（14.83-60.55）	220.69（102.07-421.75）	25.74（11.79-51.18）	-0.81 (-1.06 to -0.57)
Greece	176.79（146.99-211.14）	19.72（16.38-23.54）	437.46（355.29-533.08）	31.05（25.31-37.73）	1.63 (0.61 to 2.66)
Greenland	0.09（0.04-0.13）	5.06（2.22-7.5）	0.17（0.07-0.26）	3.4（1.47-5.14）	-1.18 (-2.7 to 0.37)
Grenada	2.68（1.83-3.46）	69.39（47.46-89.53）	3.48（2.77-4.26）	58.11（46.38-71.18）	-0.44 (-2.92 to 2.1)

Guam	0.15（0.07-0.5）	3.27（1.7-10.78）	1.79（0.72-2.62）	13.81（5.61-20.16）	4.67 (2.35 to 7.05)
Guatemala	24.17（18.83-31.08）	13.33（10.16-17.13）	53.51（41.5-67.56）	8.72（6.77-10.98）	-1.57 (-2.56 to -0.58)
Guinea	80.34（36.82-162.55）	40.2（18.32-81.23）	125.87（59.13-271.56）	39.04（18.33-83.63）	-0.08 (-0.2 to 0.04)
Guinea-Bissau	18.55（9.13-34.34）	81.23（39.66-150.86）	20.09（10.51-35.88）	58.66（30.24-105.08）	-1.04 (-1.08 to -1)
Guyana	0.19（0.14-0.25）	0.95（0.68-1.27）	16.57（10.73-24.56）	45.76（29.7-67.81）	13.29 (11.11 to 15.51)
Haiti	74.63（29.27-181.29）	40.92（15.88-98.61）	159.68（60.17-374.07）	42.25（15.86-98.8）	0.13 (-0.03 to 0.28)
Honduras	15.22（7.23-29.55）	13.42（6.34-26.08）	92.08（46.73-168.02）	26.32（13.35-48.01）	2.24 (2.07 to 2.42)
Hungary	306.56（227.95-392.92）	39.34（29.22-50.52）	606.85（449.89-797.52）	58.09（42.96-76.44）	1.23 (0.55 to 1.92)
Iceland	5.22（3.91-6.77）	30.98（23.19-40.22）	11.99（8.62-16.23）	32.49（23.36-44.01）	0.31 (-0.48 to 1.1)
India	5849.31（4267.88-8115.9）	22.98（16.67-32.03）	27923.98（15231.27-36438.07）	42.32（23.11-55.21）	2.05 (1.57 to 2.54)
Indonesia	817.92（515.51-1161.99）	16.31（10.27-23.02）	4356.93（1851.89-6490.3）	34.4（14.83-51）	2.44 (2.35 to 2.53)
Iran (Islamic Republic of)	187.7（127.22-315.05）	11.1（7.37-19.62）	649.74（443.59-1032.11）	14.31（9.76-23.26）	0.81 (0.52 to 1.1)
Iraq	318.28（165.82-557.56）	73.45（38.26-128.62）	968.07（498.05-1619.06）	80.01（41.19-134.24）	0.31 (-0.06 to 0.68)
Ireland	53.32（39.85-69.62）	22.46（16.77-29.35）	91.95（64.39-125.99）	18.85（13.22-25.8）	-0.51 (-1.73 to 0.73)
Israel	151.03（109.63-204.22）	52.6（38.25-71.01）	348.26（245.22-471.44）	47.36（33.33-64.16）	-0.33 (-0.95 to 0.28)
Italy	490.02（442.52-541.52）	9.97（8.98-11.03）	2799.9（2383.4-3203.36）	33.4（28.6-38.09）	4.06 (2.41 to 5.74)
Jamaica	67.34（45.18-94）	63.67（42.67-88.87）	137.13（88.9-201.46）	74.8（48.48-109.95）	0.62 (-0.58 to 1.84)
Japan	846.47（789.24-908.33）	9.54（8.85-10.25）	1962.17（1745.54-2173.82）	9.02（8.09-9.98）	-0.32 (-1.35 to 0.71)
Jordan	28.95（14.82-52.18）	41.97（21.18-75.9）	139.29（71.19-248.19）	34.59（17.63-61.81）	-0.61 (-0.85 to -0.37)
Kazakhstan	38.92（25.77-54.39）	7.69（4.98-10.86）	72.2（50.77-99）	8.82（6.16-12.13）	0.39 (-0.28 to 1.06)
Kenya	817.54（535.92-1578.41）	186.14（122.17-358.07）	3258.78（1752.7-6201.94）	296.37（159.97-559.57）	1.52 (1.44 to 1.6)
Kiribati	0.62（0.32-1.06）	33.62（17.4-58.02）	1.26（0.61-2.23）	36.47（17.71-64.67）	0.28 (0.17 to 0.39)
Kuwait	9.06（6.46-12.37）	28.02（19.82-38.63）	28.22（19.18-40.83）	17.52（11.89-25.35）	-0.72 (-2.27 to 0.86)

Kyrgyzstan	13.81（9.92-18.99）	11.08（7.97-15.17）	83.82（58.89-115.89）	37.51（26.24-51.87）	3.21 (-0.4 to 6.97)
Lao People's Democratic Republic	29.65（13.17-59.03）	25.87（11.46-51.4）	85.24（36.09-160.06）	35.33（15.02-66.04）	1.02 (0.91 to 1.13)
Latvia	9.06（6.54-12.04）	6.01（4.34-8）	31.72（21.88-44.13）	16.91（11.66-23.5）	3.5 (2.29 to 4.73)
Lebanon	108.64（54.4-194.57）	90.08（45.02-160.63）	291.58（143.28-522.37）	83.28（41.19-148.93）	-0.24 (-0.54 to 0.06)
Lesotho	23.21（11.71-42.14）	68.49（34.25-125.12）	49.82（24.12-87.74）	108.94（52.5-192.65）	1.56 (1.24 to 1.87)
Liberia	41.03（20.11-77.14）	56.97（27.63-107.34）	52.58（21.78-121.37）	48.79（20.23-112.12）	-0.54 (-0.75 to -0.32)
Libya	25.88（12.72-47.02）	22.98（11.25-42.14）	79.27（32.35-151.7）	28.8（11.7-55.3）	0.78 (0.24 to 1.32)
Lithuania	29.33（21.61-38.85）	14.13（10.41-18.71）	54.11（38.38-73.56）	19.75（14.03-26.83）	1.03 (-0.01 to 2.07)
Luxembourg	7.98（6.65-9.43）	28.76（23.95-33.95）	20.8（16.7-25.37）	33.62（26.99-41）	0.64 (-1.14 to 2.45)
Madagascar	515.66（255.93-970.86）	176.25（87.81-331.91）	906.6（418.28-1745.8）	171.86（79.9-330.52）	-0.1 (-0.35 to 0.15)
Malawi	485.05（223.65-911.04）	246.26（113.23-466.76）	1043.24（444.12-2032.46）	308.26（130.87-603.06）	0.76 (0.62 to 0.9)
Malaysia	86.31（22.23-162.85）	17.26（4.44-32.63）	382.93（87.03-710.01）	22.63（5.1-42.07）	0.81 (0.54 to 1.08)
Maldives	1.06（0.51-1.97）	18.16（8.75-33.59）	2.26（1.18-3.88）	12.77（6.65-22）	-1.2 (-1.62 to -0.78)
Mali	110.72（50.63-229.76）	49.43（22.28-101.91）	193.07（93.14-419.82）	40.18（19.18-86.5）	-0.64 (-0.75 to -0.52)
Malta	3.99（2.94-5.22）	16.83（12.38-22.02）	11.29（7.88-15.31）	18.29（12.74-24.82）	-0.02 (-1.31 to 1.29)
Marshall Islands	0.15（0.06-0.34）	16.34（6.94-36.83）	0.51（0.22-1.07）	25.54（11.02-52.75）	1.48 (1.33 to 1.63)
Mauritania	28.88（14.28-53.56）	55.97（27.42-103.9）	50.99（17.52-122.38）	41.85（14.49-98.89）	-0.93 (-1.05 to -0.81)
Mauritius	8.99（7.46-10.7）	23.31（19.32-27.8）	57.82（47.82-69.36）	52.84（43.63-63.32）	2.56 (-6.82 to 12.89)
Mexico	234.25（220.8-247.57）	10.01（9.42-10.59）	1205.41（993.1-1442.97）	16.9（13.94-20.19）	1.73 (0.09 to 3.39)
Micronesia (Federated States of)	0.53（0.25-1.05）	18.03（8.56-35.74）	1.29（0.61-2.35）	30.56（14.69-55.37）	1.72 (1.62 to 1.81)
Monaco	2.67（1.35-4.59）	67.24（34.03-115.62）	3.74（1.97-6.55）	62.32（32.89-108.9）	-0.26 (-0.35 to -0.16)
Mongolia	2.1（1.05-4.08）	4.01（1.99-7.82）	6.91（3.77-11.64）	6.94（3.78-11.69）	1.75 (1.11 to 2.39)
Montenegro	9.32（4.66-17.17）	29.36（14.75-54.11）	25.28（12.14-45.08）	46.21（22.24-82.2）	1.65 (1.43 to 1.87)

Morocco	158.41（77.67-286.93）	18.27（8.92-33.34）	562.59（284.82-986.38）	26.64（13.44-47.2）	1.23 (1.15 to 1.31)
Mozambique	756.17（371.62-1425.07）	249.74（122.13-472.56）	1851.77（919.83-3285.76）	370.9（182.91-666.45）	1.32 (1.2 to 1.43)
Myanmar	283.08（134.23-519.7）	22.63（10.73-41.59）	840.78（359.5-1524.53）	34.98（15.03-63.43）	1.41 (1.33 to 1.49)
Namibia	44.35（24.58-77.34）	136.04（73.8-238.98）	106.4（57.79-184.21）	178.18（96.48-309.71）	0.9 (0.79 to 1)
Nauru	0.07（0.03-0.14）	24.62（11.67-46.25）	0.11（0.05-0.21）	41.31（18.38-77.15）	1.69 (1.61 to 1.77)
Nepal	112.66（54.76-223.81）	22.1（10.67-43.91）	594.15（286.52-1101.86）	46.72（22.56-86.7）	2.46 (2.32 to 2.6)
Netherlands	242.99（187.6-312.5）	23.05（17.77-29.67）	564.8（408.73-748.81）	26.11（18.87-34.66）	0.43 (-0.15 to 1.02)
New Zealand	92.86（69.05-122.05）	41.64（30.94-54.72）	127.89（91.94-171.22）	24.53（17.63-32.84）	-0.79 (-2.95 to 1.41)
Nicaragua	12.04（7.19-17.56）	15.13（9.08-22.04）	37.56（24.3-56.72）	14.61（9.46-22.07）	-0.16 (-0.42 to 0.11)
Niger	65.93（29.71-138.72）	40.34（17.92-84.59）	124.69（49.4-293.64）	28.92（11.57-67.12）	-1.07 (-1.17 to -0.97)
Nigeria	1258.52（700.52-2004.34）	50.64（28-79.08）	1754.15（958.69-3429.75）	41.59（22.57-79.04）	-0.64 (-0.7 to -0.57)
Niue	0.02（0.01-0.04）	18.53（9.43-32.79）	0.04（0.02-0.08）	34.93（17.64-61.15）	2.08 (1.96 to 2.19)
North Macedonia	52.09（36.24-79.81）	49.98（34.82-76.88）	148.29（84.57-219.57）	77.14（44.28-113.6）	1.52 (1.24 to 1.79)
Northern Mariana Islands	0.06（0.02-0.19）	7.23（2.92-23.53）	0.31（0.15-0.79）	10.3（4.84-26.58）	1.18 (0.78 to 1.59)
Norway	92.74（82.01-104.14）	23.76（20.97-26.7）	125.74（106.3-147.1）	20.21（17.07-23.64）	-0.16 (-1.96 to 1.66)
Oman	16.45（6.71-32.71）	46.59（18.6-92.7）	49.34（22.98-90.94）	57.4（25.96-105.87）	0.71 (-0.05 to 1.48)
Pakistan	2159.84（1338.69-3371.36）	59.05（36.35-92.63）	5394.45（3142.11-8531.06）	81.24（47.42-128.56）	1.04 (0.92 to 1.15)
Palau	0.11（0.05-0.19）	17.76（8.75-32.54）	0.26（0.13-0.45）	17.94（9.19-32.14）	-0.03 (-0.14 to 0.09)
Palestine	15.63（7.79-28.22）	34.46（17.14-62.43）	40.78（22.57-69.77）	32.46（17.85-55.69）	-0.24 (-0.7 to 0.22)
Panama	18.92（15.44-22.92）	21.28（17.33-25.79）	43.38（31.09-56.66）	16.46（11.81-21.49）	-0.88 (-1.44 to -0.32)
Papua New Guinea	7.96（2.66-18.89）	7.6（2.54-18.04）	32.72（10.79-79.98）	11.27（3.72-27.48）	1.32 (1.12 to 1.52)
Paraguay	7.78（4.15-13.28）	6.22（3.3-10.6）	39.78（20.05-69.27）	11.69（5.9-20.35）	2.1 (1.63 to 2.57)
Peru	107.51（56.35-187.47）	15.95（8.35-27.83）	389.68（200.58-671.05）	20.05（10.32-34.55）	1.05 (-0.09 to 2.2)
Philippines	439.94（278.97-	29.38（18.6-	1802.31（1107.14-	42.63（26.02-	1.21

	566.97）	37.96）	2394.07）	56.22）	(1.09 to 1.33)
Poland	504.66（470.09-540.65）	23.28（21.59-24.99）	1680.12（1458.16-1898.53）	42.04（36.43-47.52）	1.75 (-0.48 to 4.03)
Portugal	251.31（191.14-327.49）	33.28（25.23-43.49）	587.7（423.94-801.12）	42.22（30.57-57.31）	0.8 (-0.4 to 2.01)
Puerto Rico	39.16（28.15-52.92）	18.57（13.35-25.06）	164.81（112.99-228.69）	39.28（26.95-54.53）	2.63 (-0.62 to 5.99)
Qatar	1.1（0.53-2.03）	21.99（10.59-40.98）	6.4（3.02-12.93）	16.47（7.84-32.99）	-0.92 (-3.18 to 1.4)
Republic of Korea	195.55（104.01-322.47）	16.2（8.58-26.85）	343.96（195.79-585.51）	6.57（3.72-11.2）	-2.87 (-3.06 to -2.68)
Republic of Moldova	120.32（96.24-148.05）	56.43（45.15-69.35）	131.42（106.58-160.32）	41.07（33.32-50.05）	-1.09 (-3 to 0.87)
Romania	612.98（421.64-843.92）	39.97（27.53-55.04）	950.24（678.41-1285.65）	45.48（32.48-61.58）	0.27 (-0.47 to 1.01)
Russian Federation	4696.15（4383.39-5031.98）	64.77（60.14-69.68）	3644.36（3134.62-4141.81）	30.05（25.86-34.15）	-2.34 (-3.59 to -1.08)
Rwanda	460.28（213.43-856.03）	322.17（149.23-599.72）	716.75（295.23-1456.47）	262.54（108.38-529.99）	-0.65 (-0.74 to -0.56)
Saint Kitts and Nevis	5.09（4.09-6.14）	237.75（189.85-288.32）	9.88（7.1-14.07）	270.32（196.14-378.94）	0.42 (-1.45 to 2.32)
Saint Lucia	2.77（2.21-3.4）	61.62（48.82-75.9）	15.87（12.1-20.47）	115.93（88.49-149.33）	2.31 (-0.74 to 5.46)
Saint Vincent and the Grenadines	0.48（0.39-0.58）	12.52（10.3-15.03）	5.7（4.61-6.93）	63.41（51.39-77.02）	5.45 (2.83 to 8.14)
Samoa	1.02（0.52-1.76）	20.5（10.42-35.7）	2.19（1.12-3.87）	26.48（13.46-47.07）	0.84 (0.7 to 0.98)
San Marino	0.17（0.09-0.29）	8.28（4.51-13.9）	0.2（0.1-0.39）	4.29（1.97-8.21）	-2.42 (-2.81 to -2.02)
Sao Tome and Principe	1.77（0.87-3.52）	51.82（25.06-101.8）	2.86（1.27-5.79）	51.67（22.91-103.98）	-0.02 (-0.42 to 0.37)
Saudi Arabia	84.79（41.86-155.42）	25.23（12.44-46.44）	336.47（153.78-633.16）	34.34（15.85-64.32）	0.99 (0.88 to 1.09)
Senegal	102.62（49.31-202.47）	54.02（25.84-106.31）	193.74（87.43-410.21）	47.4（21.55-98.97）	-0.45 (-0.65 to -0.25)
Serbia	364.67（200.31-604.51）	63.44（34.45-105.63）	645.28（350.53-1093.82）	65.43（35.44-111.11）	0.18 (0.03 to 0.33)
Seychelles	2.25（1.15-4.02）	74.62（37.84-133.93）	4.13（2.19-7.19）	67.26（35.34-117.67）	-0.36 (-0.99 to 0.27)
Sierra Leone	60.52（28.28-116.11）	49（22.84-94.22）	76.66（37.12-155.18）	37.67（18.25-75.84）	-0.83 (-0.93 to -0.74)
Singapore	11.65（8.81-15.3）	10.17（7.64-13.39）	32.8（23.89-44.33）	6.34（4.58-8.61）	-1.67 (-3.03 to -0.3)
Slovakia	118.15（68.6-192.17）	36.6（21.21-59.73）	217.52（112.72-378.46）	41.28（21.42-71.9）	0.45 (0.11 to 0.79)
Slovenia	19.41（14.63-25.23）	16.62（12.53-21.58）	98.23（69.92-134.15）	38.19（27.19-52.16）	2.7 (1.88 to 3.52)

Solomon Islands	0.97（0.35-2.19）	10.94（3.94-24.75）	3.51（1.44-7.77）	18.85（7.74-41.66）	1.77 (1.52 to 2.01)
Somalia	287.73（131.28-557.99）	282.45（129.41-549.39）	703.74（329.69-1435.42）	277.77（128.9-569.42）	-0.04 (-0.12 to 0.05)
South Africa	374.65（270.98-559.57）	40.29（29.08-59.7）	1336.61（932.93-1732.2）	63.95（44.35-82.44）	1.46 (1.08 to 1.85)
South Sudan	453.16（213.24-876.41）	248.78（117.03-482.52）	604.3（254.45-1165.38）	297.53（126.64-575.77）	0.58 (0.48 to 0.68)
Spain	702.11（550.97-883.61）	22.96（17.97-28.96）	1706.21（1221.48-2307.39）	30.6（21.97-41.26）	1.08 (0.28 to 1.88)
Sri Lanka	167.67（89.03-282.34）	27.74（14.69-47.07）	463.35（210.8-837.73）	31.13（14.38-56.21）	0.31 (-0.53 to 1.16)
Sudan	96.72（39.38-209.61）	16.27（6.59-35.4）	250.62（125.34-445.83）	21.19（10.56-38.08）	0.85 (0.78 to 0.92)
Suriname	5.65（3.06-9.53）	39.07（21.13-65.96）	18.97（9.23-34.03）	54.01（26.14-97.45）	1.15 (0.34 to 1.97)
Sweden	155.72（118.06-199.66）	17.92（13.63-22.91）	168.71（118.93-231.55）	12.43（8.8-16.97）	-0.86 (-1.86 to 0.16)
Switzerland	145.51（105.39-193.19）	26.7（19.37-35.41）	140.83（97.16-197.44）	12.98（8.98-18.14）	-2.44 (-3.32 to -1.55)
Syrian Arab Republic	6.92（3.5-12.94）	2.16（1.09-4.01）	22.74（11.4-40.86）	2.83（1.41-5.1）	0.86 (0.55 to 1.17)
Taiwan (Province of China)	99.64（76.74-126.79）	8.82（6.76-11.26）	431.52（316.36-574.64）	15.88（11.61-21.18）	1.54 (0.49 to 2.6)
Tajikistan	5.94（2.56-12.58）	4.54（1.95-9.71）	18.74（7.76-40.88）	6.35（2.66-13.57）	0.99 (0.43 to 1.55)
Thailand	216.28（115.77-370.25）	12.35（6.58-21.19）	823.74（418.54-1375.12）	12.9（6.56-21.53）	0.15 (-0.17 to 0.47)
Timor-Leste	1.78（0.83-3.4）	13.6（6.29-26.01）	12.24（5.18-23.2）	24.2（10.22-45.86）	1.88 (1.8 to 1.96)
Togo	32.16（15.18-64.38）	50.64（23.66-101.44）	83.76（42-161.64）	51.19（25.52-98.54）	0.04 (-0.08 to 0.17)
Tokelau	0.01（0.01-0.03）	13.41（5.79-27.29）	0.02（0.01-0.04）	23.22（11.12-43.56）	1.8 (1.72 to 1.88)
Tonga	0.43（0.21-0.83）	13.33（6.39-25.85）	1.16（0.55-2.11）	26.02（12.39-47.35）	2.2 (1.79 to 2.61)
Trinidad and Tobago	44.65（37.09-53.85）	95.06（78.85-114.7）	85.7（60.35-118.59）	70.92（50.16-97.69）	-0.84 (-2.74 to 1.1)
Tunisia	166.67（83.39-301.34）	55.11（27.61-99.38）	532.44（226.85-1000.18）	68.1（29.24-127.67）	0.69 (0.53 to 0.84)
Turkey	731.59（375.67-1364.94）	39.83（20.33-74.49）	1947.19（1031.54-3303.3）	36.47（19.36-62.2）	-0.33 (-0.55 to -0.11)
Turkmenistan	10.55（7.27-14.21）	12.7（8.6-17.29）	40.95（25.97-62.77）	21.74（13.91-33.04）	1.76 (1.5 to 2.02)
Tuvalu	0.06（0.02-0.13）	14.39（6-32.08）	0.15（0.07-0.28）	24.99（11.66-46.75）	1.81 (1.73 to 1.88)

Uganda	1114.28（590.52-1906.45）	320.44（169.22-549.61）	2477.16（1241.42-4374.3）	391.86（197.38-693.46）	0.64 (0.52 to 0.77)
Ukraine	1315（927.79-1785.08）	39.26（27.69-53.41）	1329.86（777.06-2062.12）	32.89（19.32-50.92）	-0.6 (-1.27 to 0.08)
United Arab Emirates	2.85（1.21-6.15）	14.4（6.14-30.92）	24.64（12.87-43.76）	11.64（6.03-20.95）	-0.94 (-1.74 to -0.14)
United Kingdom	1709.34（1620.87-1796.89）	34.77（32.83-36.6）	1432.64（1308.57-1541.91）	17.94（16.42-19.31）	-2.14 (-2.56 to -1.72)
United Republic of Tanzania	1514.26（745.11-2789.34）	251.5（123.28-465.7）	3026.19（1342.76-6197.37）	235.18（104.83-481.08）	-0.21 (-0.3 to -0.12)
United States of America	2.06（1.04-3.98）	40.53（37.75-43.4）	6.77（3.43-12.2）	32.2（29.39-34.97）	0.96 (0.09 to 1.84)
United States Virgin Islands	7155.69（6685.44-7653.39）	48.08（24.32-93.18）	11545.78（10558.89-12530.58）	62.89（31.87-113.21）	-0.66 (-1.41 to 0.1)
Uruguay	191.29（137.3-257.26）	86.26（61.83-116.2）	188.13（136.94-256.48）	62.88（45.79-85.7）	-1.28 (-2.74 to 0.19)
Uzbekistan	22.28（11.72-33.29）	4.44（2.26-6.66）	70.64（50.52-98.17）	5.54（3.96-7.71）	0.76 (-1.11 to 2.65)
Vanuatu	0.44（0.17-0.99）	11.26（4.38-25.19）	2.02（0.87-4.18）	20.37（8.71-42.31）	1.93 (1.75 to 2.11)
Venezuela (Bolivarian Republic of)	76.24（62.61-90.92）	14.46（11.88-17.26）	374.3（260.57-513.35）	21.53（15.04-29.39）	1.35 (-0.21 to 2.94)
Viet Nam	696.19（379.61-1168.62）	35.53（19.22-59.76）	2656.25（1378.3-4592.3）	56.62（29.01-97.8）	1.55 (1.43 to 1.67)
Yemen	47.57（17.72-108.02）	17.07（6.36-38.86）	127.37（51.92-274.07）	16.36（6.66-35.36）	-0.13 (-0.27 to 0)
Zambia	447.28（223.59-814.83）	274.24（136.36-501.88）	1121.17（419.46-2901.27）	350.76（133.32-876.99）	0.82 (0.6 to 1.03)
Zimbabwe	64.98（33.93-114.59）	31.57（16.46-55.6）	137.36（73.87-235.78）	49.86（26.19-86.62）	1.55 (1.32 to 1.79)

Abbreviations: SDI: Sociodemographic Index; ASDR: age-standardised disability-adjusted life years rate; DALYs: disability-adjusted life years; AAPC: average annual percentage change; CI: confidence interval; UI: uncertainty interval.

Table S4. Changes in incident number according to population-level determinants and causes from 1990 to 2021.

Location	Overall difference [a]	Change due to population-level determinants			Percent change of Aging	Percent change of Population	Percent change of Epidemiological change	Overall percent change
		Aging [b]	Population [c]	Epidemiological change [d]				
Global	17844.37	337.5	10483.13	7023.75	5.82	180.8	121.14	307.75
High SDI	3273.92	114.63	2703.18	456.11	4.89	115.27	19.45	139.61

High-middle SDI	4950.47	91.47	2490.32	2368.68	6.82	185.63	176.56	369.01
Low SDI	877.16	23.29	777.16	76.7	3.47	115.62	11.41	130.49
Low-middle SDI	1870.87	28.96	1039.45	802.46	5.31	190.64	147.18	343.13
Middle SDI	6858.01	50.33	3214.87	3592.81	5.66	361.82	404.35	771.83

a.Change in incident number between year 2021 and 1990;

b.Change in incident number due to change in the age structure;

c.Change in incident number due to change in population number;

d.Change in incident number due to epidemiologic changes. Epidemiologic changes refer to the incident number change when age structure and population hold constant.

Abberrations: SDI = Socio-demographic index.

Table S5. Changes in death number according to population-level determinants and causes from 1990 to 2021.

Overall difference [a]	Change due to population-level determinants (% contribute to the total changes)			Percent change of Aging	Percent change of Population	Percent change of Epidemiological change	Overall percent change
	Aging [b]	Population [c]	Epidemiological change [d]				

5924.18	357.89	4634.94	931.35	11.33	146.7	29.48	187.5
744.99	120.02	799.78	-174.81	15.7	104.59	-22.86	97.43
1070.19	87.9	896.9	85.39	12.43	126.85	12.08	151.36
626.29	32.14	658.73	-64.58	5.29	108.4	-10.63	103.06
1256.78	36.98	802.28	417.52	7.74	167.83	87.34	262.91
2220.06	83.95	1403.06	733.06	14.03	234.43	122.48	370.94

a.Change in death number between year 2021 and 1990;

b.Change in death number due to change in the age structure;

c.Change in death number due to change in population number;

d.Change in death number due to epidemiologic changes. Epidemiologic changes refer to the death number change when age structure and population hold constant.

Abberrations: SDI = Socio-demographic index.

Table S6. Changes in DALYs number according to population-level determinants and causes from 1990 to 2021.

Overall difference [a]	Change due to population-level determinants (% contribute to the total changes)						
	Aging [b]	Population [c]	Epidemiological change [d]	Percent change of Aging	Percent change of Population	Percent change of Epidemiological change	Overall percent change

5924.18	357.89	4634.94	931.35	11.33	146.7	29.48	187.5
744.99	120.02	799.78	-174.81	15.7	104.59	-22.86	97.43
1070.19	87.9	896.9	85.39	12.43	126.85	12.08	151.36
626.29	32.14	658.73	-64.58	5.29	108.4	-10.63	103.06
1256.78	36.98	802.28	417.52	7.74	167.83	87.34	262.91
2220.06	83.95	1403.06	733.06	14.03	234.43	122.48	370.94

a.Change in DALYs number between year 2021 and 1990;

b.Change in DALYs number due to change in the age structure;

c.Change in DALYs number due to change in population number;

d.Change in DALYs number due to epidemiologic changes. Epidemiologic changes refer to the DALYs number change when age structure and population hold constant.

Abberrations: SDI = Socio-demographic index.
